# Skeletocutins M–Q: biologically active compounds from the fruiting bodies of the basidiomycete *Skeletocutis* sp. collected in Africa

**DOI:** 10.3762/bjoc.15.270

**Published:** 2019-11-19

**Authors:** Tian Cheng, Clara Chepkirui, Cony Decock, Josphat C Matasyoh, Marc Stadler

**Affiliations:** 1 Department of Microbial Drugs, Helmholtz Centre for Infection Research (HZI), German Centre for Infection Research (DZIF), Partner Site Hannover/Braunschweig, Inhoffenstraße 7, 38124 Braunschweig, Germany; 2 Mycothéque de l’ Universite Catholique de Louvain (BCCM/MUCL), Place Croix du Sud 3, B-1348 Louvain-la-Neuve, Belgium; 3 Department of Chemistry, Faculty of Science, Egerton University, P.O. Box 536, 20115, Egerton, Kenya

**Keywords:** basidiomycete, polyporaceae, secondary metabolites, structure elucidation

## Abstract

During the course of screening for new metabolites from basidiomycetes, we isolated and characterized five previously undescribed secondary metabolites, skeletocutins M–Q (**1**–**5**), along with the known metabolite tyromycin A (**6**) from the fruiting bodies of the polypore *Skeletocutis* sp. The new compounds did not exhibit any antimicrobial, cytotoxic, or nematicidal activities. However, compound **3** moderately inhibited the biofilm formation of *Staphylococcus aureus* (*S. aureus*), while compounds **3** and **4** performed moderately in the ʟ-leucine-7-amido-4-methylcoumarin (ʟ-Leu-AMC) inhibition assay. These compounds represent the first secondary metabolites reported to occur in the fruiting bodies by *Skeletocutis*. Interestingly, tyromycin A (**6**) was found to be the only common metabolite in fruiting bodies and mycelial cultures of the fungus, and none of the recently reported skeletocutins from the culture of the same strain were detected in the basidiomes.

## Introduction

Over the past years, we have been studying the secondary metabolites of African Basidiomycota that were collected in rainforests and mountainous areas of Western Kenya. These species were new to science, and proved to be a prolific source of unprecedented natural compounds showing a set of prominent biological activities [1–3].

The present study deals with the comparison of the secondary metabolites located in the basidiomes (fruiting bodies) of another, putatively new species belonging to the genus *Skeletocutis*, strain MUCL56074. We have recently reported the known metabolite tyromycin A (**6**), together with 12 unprecedented congeners for which we proposed the trivial names skeletocutins A–L, which were obtained from a liquid culture of the same fungus [4]. A preliminary characterization of the producer organism suggested that it belongs to a new species because neither DNA sequence data in the public domain nor morphological characteristics matched the previously reported species, as compared to the literature. The genus *Skeletocutis* (of the Polyporaceae) consists of approximately 40 species, which grow as a crust on the surface of collapsing wood [5] and mostly occur in the temperate climate zones.

In our preceding study, the fungal specimen MUCL56074 has been assigned to the genus *Skeletocutis* by comparison of morphological features and 5.8S/ITS rDNA sequences, as reported previously [4]. Strain MUCL56074 represents a hitherto undescribed species, which will be formally described in a separate paper in a mycological journal, pending the examination of type material of related species. In view of a potential application of chemotaxonomic methodology, the basidiomes of the fungus were checked for the presence of secondary metabolites for later comparison with herbarium specimens of other species by HPLC–diode array detection (HPLC–DAD)–MS. Surprisingly, we detected further members of the skeletocutin family that were not present in the cultures. The current paper is dedicated to the description of their isolation as well as biological and physicochemical characterization.

## Results and Discussion

**Figure 1:**
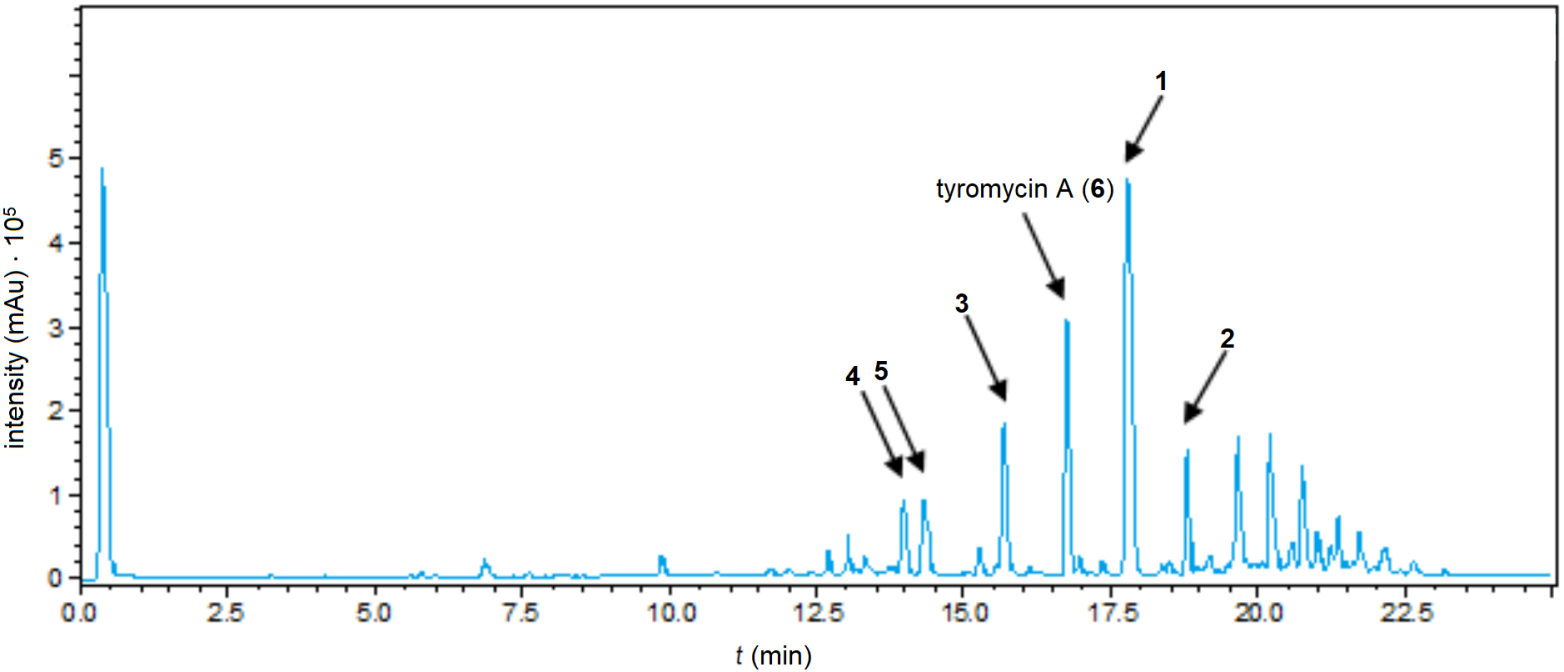
HPLC–UV chromatogram of the extract from fruiting bodies of *Skeletocutis sp.* (detection wavelength λ = 190–600 nm).

The fruiting bodies of the fungal specimen MUCL56074 were extracted with acetone and subsequently purified via preparative HPLC, which led to the isolation of five previously undescribed secondary metabolites, **1**–**5** (*t_R_* = 17.8, 18.8, 15.7, 14.0, and 14.3 min, respectively), and one known compound, namely tyromycin A (**6**, *t_R_* = 16.8 min) [6] (Figure 1).

**Figure 2:**
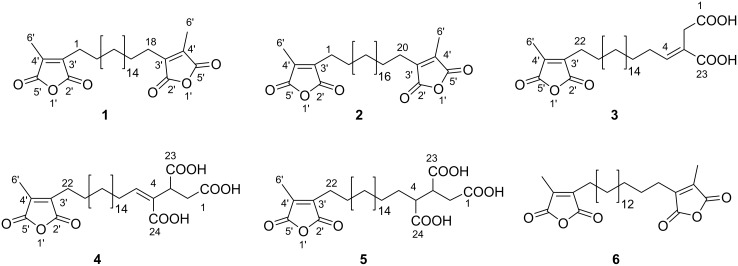
Chemical structures of compounds **1**–**6**.

Compound **1** (Table 1 and Figure 2), named skeletocutin M, was isolated as a yellow solid. Its molecular formula was determined to be C_28_H_42_O_6_, with eight degrees of unsaturation, by HRESIMS. Signals for *m*/*z* = 475.3054, 497.2868, and 971.5839, corresponding to the ions [M + H]^+^, [M + Na]^+^, and [2M + Na]^+^, respectively, were also recorded in the mass spectrum. A singlet resonating at δ = 2.08 ppm for the methyl protons H_3_-6′ and a triplet and quinted resonating at δ = 2.50 and 1.59 ppm, respectively, for the methylene groups, were recorded in the ^1^H NMR spectrum. Further, the ^13^C NMR spectrum revealed only 14 signals instead of 28, as indicated by the molecular formula, suggesting that the molecule consisted of two identical halves.

**Table 1:** ^1^H and ^13^C NMR data for **1** (in acetone-*d*_6_) and **2** (in CDCl_3_).

	**1**		**2**	

Position	^13^C/DEPT	^1^H	^13^C/DEPT	^1^H

1/18 (**1**) or 1/20 (**2**)	24.8, CH_2_	2.50 (t), *J* = 7.6 Hz	24.4, CH_2_	2.46 (t), *J* = 7.6 Hz
2/17 (**1**) or 2/19 (**2**)	28.2, CH_2_	1.59 (p), *J* = 7.6 Hz	27.6, CH_2_	1.58 (p), *J* = 7.6 Hz
3–16 (**1**) or 3–18 (**2**)	29.4–29.8, CH_2_	1.28–1.36 (m)	29.2–29.7, CH_2_	1.26–1.32 (m)
2’	167.1, C		165.9, C	
3’	144.9, C		144.8, C	
4’	141.7, C		140.4, C	
5’	167.4, C		166.3, C	
6’	9.6, CH_3_	2.08 (s)	9.5, CH_3_	2.08 (s)

Determination of HMBC correlations between the C-6′ methyl protons (δ = 2.08 ppm) and C-3′/4′/5′ as well as between H_2_-1/18 and C-2′/3′/4′ confirmed the presence of a maleic anhydride moiety in the molecule. Integration of the singlet for the C-6′ methyl group in the ^1^H NMR spectrum gave a value of 6, indicating the presence of two maleic anhydride functions. The multiplet at δ = 1.28–1.36 ppm was assigned to the remaining 14 methylene units making up the carbon chain. Integration of this multiplet gave a value of 28, confirming the length of the chain. The connection of this chain to two maleic anhydride units was confirmed by HMBC correlations between the protons H_2_-1/2/17/18 and C-3′ (δ = 144.9 ppm). Additionally, long-range correlations between the protons H_2_-1 and H_3_-6′ were observed in the COSY spectrum. Therefore, the structure of natural product **1** was unambigously concluded to be 1,18-bis[4′-methyl-2′,5′-dioxo-3′-furyl]octadecane.

Compound **2** (skeletocutin N, Table 1 and Figure 2) was obtained as a white solid, with the molecular formula C_30_H_46_O_6_ and eight degrees of unsaturation determined from HRESIMS data. The 1D and 2D NMR data for **2** revealed a similar structure to **1**, with the difference being the size of the carbon chain in the molecule. A value of 32 obtained from the integration of the C-3–C-18 multiplet (δ = 1.26–1.32 ppm) led to the conclusion that **2** had an icosane chain instead of the octadecane chain elucidated for skeletocutin M (**1**).

Compound **3** (skeletocutin O, Table 2 and Figure 2), isolated as a yellow oil, had the molecular formula C_28_H_44_O_7_ and seven degrees of unsaturation, deduced from HRESIMS data. Signals for *m*/*z* = 493.3163 ([M + H]^+^), 515.2980 ([M + Na]^+^), 475.3058 ([M + H − H_2_O]^+^), and 1007.6067 ([2M + Na]^+^) were also recorded by HRESIMS. A singlet for a methyl group (δ = 2.08 ppm, H_3_-6′), along with another singlet (δ = 3.37 ppm, H_2_-2), a quintet (δ = 1.58 ppm, H_2_-21), a triplet (δ = 2.45 ppm, H_2_-22) for three methylene groups, and a triplet for a methine unit *(*δ = 7.13 ppm, H-4) were observed in the ^1^H NMR spectrum of **3**. Analysis of 1D and 2D NMR data for **3** indicated a similar structure to **1**, with a difference being the absence of one maleic anhydride moiety, which was replaced by a dicarboxylic acid motif and an olefinic bond between position C-3 (δ = 124.5 ppm) and C-4 (δ = 148.9 ppm). Resonances for the carbon atoms of the carboxylic acid moieties occurred at δ = 176.8 ppm (C-1) and 172.0 ppm (C-23). The dicarboxylic acid function could be elucidated through HMBC correlations between H_2_-2 and C-1/3/4/23, H-4 and C-23/3, as well as H_2_-5 and C-3. Further cross peaks between H_2_-5 and H_2_-6/H-4 were observed in COSY spectra, which confirmed the linkage of the dicarboxylic acid motif to the chain at C-3. The diastereotopic protons H_2_-2 (δ = 3.37) showed ROESY correlations to H_2_-5 (δ = 2.23 ppm), but no correlations between H_2_-2 and H-4 (δ = 7.13 ppm) were recorded. Therefore, the configuration of the olefinic bond between C-3 and C-4 was assigned (*E*)-configuration. As such, the structure of **3** was concluded to be (*E*)-2-(19-(4’-methyl-2’,5’-dioxo-2’,5’-dihydrofuran-3’-yl)nonadecylidene)butanedioic acid.

**Table 2:** ^1^H and ^13^C NMR data for **3** (in CDCl_3_) and compounds **4** and **5** (in DMSO-*d*_6_).

	**3**		**4**		**5**	

Position	^13^C/DEPT	^1^H	^13^C/DEPT	^1^H	^13^C/DEPT	^1^H

1	176.8, C		174.5, C		174.3, C	
2	32.1, CH_2_	3.37 (s)	35.8, CH_2_	2.23 (dd), *J* = 16.3, 5.8 Hz 2.92 (dd), *J* = 16.3, 7.8 Hz	33.6, CH_2_	2.29 (dd), *J* = 16.6, 2.4 Hz 2.53 (m)^a^
3	124.5, C		39.6, CH	3.86 (dd), *J* = 7.8, 5.8 Hz	42.8, CH	2.83 (ddd), *J* = 10.5, 6.7, 3.4 Hz
4	148.9, CH	7.13 (t), *J* = 7.6 Hz	131.9, C		46.1, CH	2.50 (m)^a^
5	29.12, CH_2_	2.23 (m)	144.7, CH	6.74 (t), *J* = 7.4 Hz	28.6, CH_2_	1.56 (m)
6	28.3, CH_2_	1.48 (m)	28.5, CH_2_	2.19 (q), *J* = 7.4 Hz	28.6, CH_2_	1.35 (m)
7			28.5, CH_2_	1.38 (m)		
8–20	29.3–29.7, CH_2_	1.26–1.31 (m)	29.1–29.5, CH_2_	1.23–1.26 (m)	28.82–29.0, CH_2_	1.23–1.26 (m)
21	27.6, CH_2_	1.58 (p), *J* = 7.6 Hz	27.3, CH_2_	1.48 (p), *J* = 7.5 Hz	26.9, CH_2_	1.49 (p), *J* = 8.0 Hz
22	24.4, CH_2_	2.45 (t), *J* = 7.6 Hz	24.1, CH_2_	2.39 (t), *J* = 7.5 Hz	23.6, CH_2_	2.40 (t), *J* = 8.0 Hz
23	172.0, C		173.8, C		172.9, C	
24			167.8, C		174.7, C	
2’	165.9, C		166.7, C		166.2, C	
3’	144.8, C		143.9, C		143.4, C	
4’	140.4, C		141.3, C		140.8, C	
5’	166.3, C		166.9, C		166.4, C	
6’	9.3, CH_3_	2.08 (s)	9.7, CH_3_	1.99 (s)	9.2, CH_3_	2.00 (s)

^a^Overlapping signals.

Compound **4** (Table 2 and Figure 2), named skeletocutin P, was isolated as a white solid. Its molecular formula was established to be C_29_H_44_O_9_, with eight degrees of unsaturation, from HRESIMS data. Peaks for *m*/*z* = 537.3058 ([M + H]^+^), 559.2877 ([M + Na]^+^), 519.2953 ([M + H − H_2_O])^+^, and 1095.5860 ([2M + Na]^+^) were observed in the mass spectrum. The 1D and 2D NMR data of **4** were similar to those of **3**, with the difference being the presence of a tricarboxylic acid moiety instead of a dicarboxylic acid motif at one end of the chain. The three carboxylic acid functions of the molecule had resonances at δ = 174.5 (C-1), 173.8 (C-23), and 167.8 ppm (C-24) in the ^13^C NMR spectrum. The HMBC correlations between H_2_-2 and C-1/3/4/23 as well as H-5 and C-3/24 and the COSY correlations between H_2_-2 and H-3 confirmed the tricarboxylic acid moiety in the molecule. COSY correlations between H_2_-6 and H_2_-5/7 confirmed the linkage of this moiety to the rest of the molecule. The absence of ROESY correlations between H-5 and H-3 but the presence of such between the protons H-3 and H_2_-6 indicated (*Z*)-configuration of the olefinic bond between C-4 and C-5. Therefore, the structure of **4** was unambiguously elucidated as (*Z*)-21-(4’-methyl-2’,5’-dioxo-2’,5’-dihydrofuran-3’-yl)henicos-3-ene-1,2,3-tricarboxylic acid.

Compound **5** (skeletocutin Q, Table 2 and Figure 2), with the molecular formula C_29_H_46_O_9_ and seven degrees of unsaturation, as established from HRESIMS data, was obtained as a yellow solid. Analysis of 1D and 2D NMR data of **5** indicated a similar structure to **4**, with saturation of the olefinic bond between C-4 and C-5. In the ^13^C NMR spectrum of **5**, the signals that had occurred at δ = 144.7 and 131.9 ppm for compound **4** were missing, and instead, a methylene signal at δ = 28.5 ppm (C-5) and a methine signal at δ = 46.1 ppm (C-4) were recorded. HMBC correlations were observed between H-4 (δ = 2.50 ppm) and C-2/3/23/24 as well as H_2_-5 (δ = 1.56 ppm) and C-4/C6/24. Furthermore, COSY correlations between H-3 and H_2_-2/H-4 as well as H_2_-5 and H-4/H_2_-6 could be recorded. Hence, **5** was concluded to be 21-(4’-methyl-2’,5’-dioxo-2’,5’-dihydrofuran-3’-yl)henicosane-1,2,3-tricarboxylic acid.

Tyromycin A (**6**), a closely related compound to the metabolites **1**–**5**, has been reported before, and was isolated from the cultures of the same fungus (i.e., the corresponding mycelial culture of the specimen that was the subject of the present study [4]) and originally from *Tyromyces lacteus* [6]. In these two cases, **6** was reported to be the major component of the culture extracts. Even though this compound is occurring in fruiting bodies, in this case, skeletocutin M (**1**) was the major component instead of tyromycin A (**6**). The two molecules **1** and **6** differ in their chain length, with the former having an 18-carbon chain instead of a 16-carbon chain, as in **6**.

**Figure 3:**
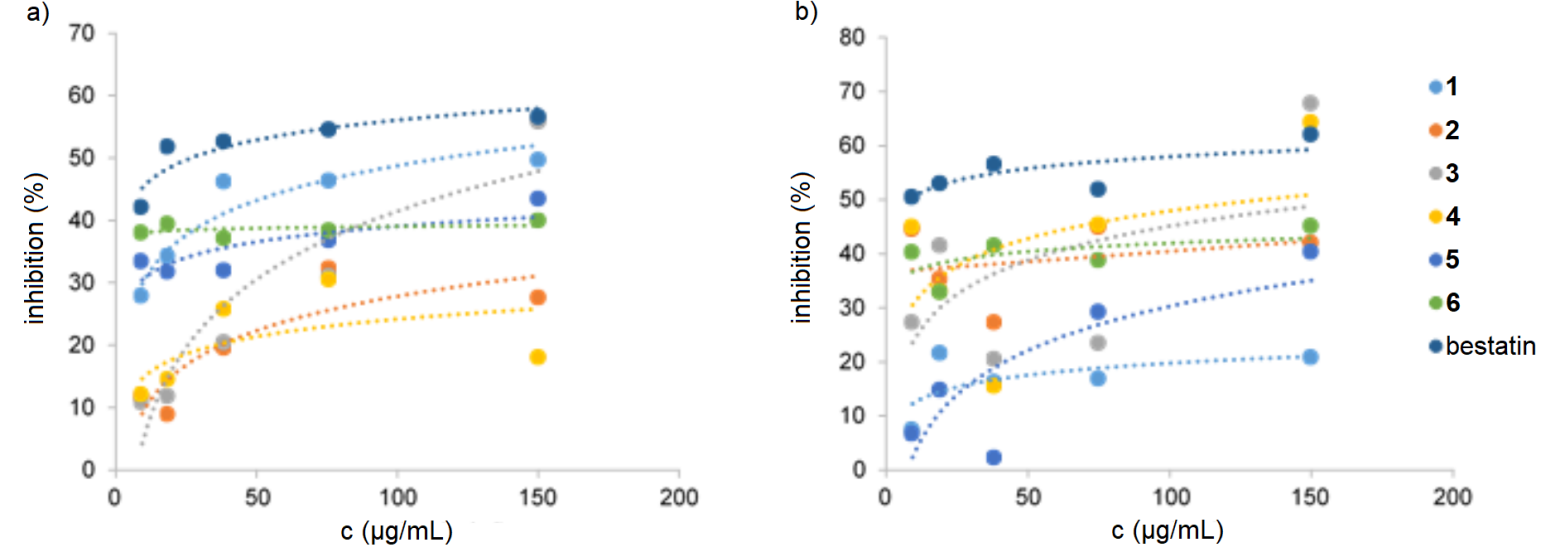
Inhibition Leu-AMC hydrolysis. a) *c* (ʟ-Leu-AMC) = 100 µM. b) *c* (ʟ-Leu-AMC) = 50 µM.

The isolated compounds **1**–**6** were evaluated for antimicrobial, cytotoxic, and nematicidal activities, as described in the Experimental section. However, compounds **1**–**5** were devoid of activity in these assays, whereas tyromycin A (**6**) and skeletocutin A–L had been reported before to be active against several Gram-positive bacteria [4], namely *Bacillus subtilis* (*B. subtilis*), *S. aureus*, methicillin-resistant *S. aureus* (MRSA), and *Micrococcus luteus* (*M. luteus*). In the antimicrobial assay, compounds **3** and **5** were observed to interfere with the formation of biofilms commonly associated with *S. aureus*. When compounds **3** and **5** were evaluated for biofilm inhibition activity against *S. aureus*, they showed only weak activity with 20 and 56% inhibition of the biofilm, respectively, at a concentration 256 µg/mL. Tyromycin A (**6**) was previously reported to be an inhibitor of leucine aminopeptidase in HeLa S3 cells [6]. Accordingly, all compounds **1**–**5** were tested for their inhibition activity against hydrolysis of ʟ-leucine-7-amido-4-methylcoumarin (ʟ-Leu-AMC). Compound **4** exhibited moderate activity, with an IC_50_ value of 71.1 µg/mL (Table 3 and Figure 3) when 50 µM of the substrate was used. Compounds **3** and **5** exhibited weak activities, with IC_50_ values of >80 µg/mL at 50 and 110 µM substrate concentration. Although tyromycin A (**6**) was previously reported to be active in a similar assay against HeLa S3 cells, with IC_50_ values of 31 μg/mL at 50 μM substrate concentration, an IC_50_ value >150 μg/mL for this compound was recorded on the HeLa (KB3.1) cells during this cytotoxicity study [6].

**Table 3:** Inhibition of ʟ-Leu-AMC hydrolysis by the metabolites **1**–**5**.

IC_50_ (μg/mL)							

Substrate (c)	**1**	**2**	**3**	**4**	**5**	**6**	Bestatin [12]

ʟ-Leu-AMC (50 µM)	–	–	89.6	71.1	153.2	138.4	10.8
ʟ-Leu-AMC (100 µM)	–	–	130.4	102.3	225.3	–	40.9

## Conclusion

In summary, five previously undescribed tyromycin A derivatives **1**–**5** could be isolated from *Skeletocutis* sp. fruiting bodies. These metabolites are closely related to the skelotocutins that were previously reported as isolates from liquid cultures. Compounds **3** and **5** were observed to weakly inhibit the biofilm formation by *S. aureus* and constrain inhibitory activity of ʟ-Leu-AMC hydrolysis in KB 3.1 cells. There have been relatively few studies on the production of secondary metabolites in mycelial cultures vs fruiting bodies in higher fungi, but so far, there are only few examples where the same compounds were predominant in both. For instance, in most species hitherto studied of the ascomycete order Xylariales, the fruiting bodies and cultures mostly showed a complementary secondary metabolite production [7]. In the current case, it appears that the basidiomes of *Skeletocutis* can be used for chemotaxonomic studies. Investigations of herbarium specimens may not even be helpful for the taxonomic revision of the genus but may even lead to the discovery of further, previously undescribed members of the tyromycin/skeletocutin type.

## Experimental

### General information

NMR spectra were recorded with a Bruker 500 MHz spectrometer at frequencies of 500.130 (^1^H NMR) and 125.758 MHz (^13^C NMR). HRESIMS spectra were recorded after purification with an Agilent 1200 series HPLC–UV system (column size: 2.1 mm⋅50 mm, packing: 1.7 µm, Waters ACQUITY UPLC BEH C18 sorbent, solvent A: H_2_O + 0.1% formic acid, solvent B: acetonitrile + 0.1% formic acid, elution gradient: 5% solvent B for 0.5 min, increasing solvent B to 100% within 19.5 min, 100% solvent B for 5 min, flow rate: 0.6 mL/min, UV–vis detection at λ = 200–600 nm) and ESI–TOF–MS analysis (maXis™ system, Bruker, scan range: 100–2500 *m*/*z*, capillary voltage: 4500 V, drying temperature: 200 °C). UV–vis spectra were recorded with a Shimadzu UV-2450 UV–vis spectrophotometer. The chromatogram in Figure 1 was recorded on a Bruker Agilent 1260 Infinity Series equipped with DAD and an ESI ion trap mass spectrometer (amaZon speed ion trap, Bruker).

### Fungal material

The fungal specimen was collected by C. Decock and J. C. Matasyoh in the Mount Elgon National Reserve [4]. The dried specimen and corresponding cultures were deposited in the MUCL collection (MUCL accession number: 56074).

### Extraction of the crude extract

A quantity of 9.8 g fruiting bodies were extracted using 500 mL of acetone overnight. Then, the extract was filtered and another 500 mL of acetone were added. This was extracted in an ultrasonic bath for 30 min. The extracts were combined and the solvent evaporated to afford 226 mg of crude extract.

### Isolation of compounds **1–6**

The crude extract (vide supra) was filtered using solid-phase microextraction (SPME) with a Strata™-X 33 µm Polymeric Reversed Phase (RP) cartridge (Phenomenex). The extract was fractionated by preparative RP chromatography (RPC) using a PLC 2020 purification system (Gilson). A VarioPrep (VP) column system packed with NUCLEODUR^®^ 100-5 C18 ec was used as stationary phase (Machery-Nagel, column size: 25 mm⋅40 mm, packing: 7 µm). Deionized water, obtained from a Milli-Q^®^ water purification system (Millipore), + 0.05% TFA (solvent A) and acetonitrile + 0.05% TFA (solvent B), respectively, were used as mobile phases (elution gradient: 50% solvent B, increasing solvent B to 100% within 60 min, 100% solvent B for 5 min, flow rate: 40 mL/min, UV–vis detection at 210, 254, and 350 nm). Eight fractions (F1–F8) were collected, in accordance with the observed signals.

F1 was further purified by RPC using solvent A and solvent B (elution gradient: 45% solvent B for 3 min, increasing solvent B to 100% within 18 min, 100% solvent B for 4 min). A Kromasil^®^ C18 HPLC column (Nouryon, column size: 250 mm⋅20 mm, packing: 7 μm, flow rate: 15 mL/min) was used as stationary phase. This led to the isolation of compound **4** (2.50 mg). Using the same column and elution gradient, **5** (2.87 mg) was purified from F2, compound **3** (4.26 mg) from F4, compound **1** (4.84 mg) from F6, compound **2** (2.26 mg) from F7, and tyromycin A (**6**, 2.15 mg) was obtained from F8.

### Physicochemical data for compounds **1–5**

Skeletocutin M (**1**): yellow solid (4.84 mg). UV (MeOH) λ_max_, (log ε): 220 nm (4.26); HRESIMS (*m/z*): [M + H]^+^ calcd for C_28_H_43_O_6_, 475.3054; found, 475.3059.

Skeletocutin N (**2**): white solid (2.26 mg). UV (MeOH) λ_max_, (log ε): 214 (4.84), 254 nm (5.02); HRESIMS (*m/z*): [M + H]^+^ calcd for C_30_H_47_O_6_, 503.3374; found, 503.3373.

Skeletocutin O (**3**): yellow oil (4.26 mg). UV (MeOH) λ_max_, (log ε): 220 nm (3.87); HRESIMS (*m/z*): [M + H]^+^ calcd for C_28_H_45_O_7_, 493.3163; found, 493.3165.

Skeletocutin P (**4**): white solid (2.50 mg). [α]_D_^20^ +12 (*c* 0.0025, MeOH); UV (MeOH) λ_max_, (log ε): 222 nm (5.14); HRESIMS (*m/z*): [M + H]^+^ calcd for C_29_H_45_O_9_, 537.3058; found, 537.3064.

Skeletocutin Q (**5**): yellow solid (2.87 mg). [α]_D_^20^ +8.7 (*c* 0.001, MeOH); UV (MeOH) λ_max_, (log ε): 206 (4.49), 256 nm (4.65); HRESIMS (*m/z*): [M + H]^+^ calcd for C_29_H_47_O_9_, 539.3215; found, 539.3220.

### Antimicrobial assay

Minimum inhibitory concentrations (MIC) were determined in serial dilution assays using several microorganisms, as described previously [8–9]. Herein, Gram-positive bacteria used were *B. subtilis* DSM10, MRSA DSM11822, *S. aureus* DSM346, *M. luteus* DSM20030, and *Mycobacterium smegmatis* (*M. smegmatis)* ATCC700084, Gram-negative bacteria were *Escherichia coli* (*E. coli*) DSM498, *Chromobacterium violaceum* (*C. violaceum*) DSM30191, and *Pseudomonas aeruginosa* (*P. aeruginosa*) PA14. Moreover, the filamentous fungus *Mucor plumbeus* (*M. plumbeus*) MUCL49355 and the yeasts *Candida tenuis* (*C. tenuis*) MUCL29892, *Pichia anomala* (*P. anomala*) DSM6766, and *Candida albicans* (*C. albicans*) DSM1665 were applied. The assays were conducted in 96-well plates and Mueller–Hinton Broth (MHB) for bacteria, or yeast, malt, and gluocse (YMG) medium for filamentous fungus and yeasts.

### Cytotoxicity assay

In vitro cytotoxicity, using IC_50_ values as a measure, was evaluated against mouse fibroblasts cell line L929 and HeLa (KB3.1) cells and carried out according to our previous reports [8–9].

### Inhibition of biofilm formation

The assay was performed in Falcon^®^ 96-well flat bottom plates as previously described [10]. *S. aureus* DSM1104 was enriched overnight to reach 0.5 McFarland standard turbidity in casein-peptone soymeal-peptone (CASO) medium containing 4% glucose at pH 7.0 for biofilm formation. Methanol was used as negative control, while tetracycline was used as positive control. All experiments were made in triplicates.

### Nematicidal activity assay

The nematicidal activity of isolated compounds against *Caenorhabditis elegans* (*C. elegans*) was performed in 24-well microtiter plates as previously described [11]. Ivermectin was used as positive control and methanol was used as negative control. The results are expressed as LD_90_ values.

### Inhibition of leucine aminopeptidases

Hydrolysis of ʟ-Leu-AMC by the surface-bound aminopeptidases of KB3.1 cells was assayed based on the method from Weber and co-workers [6] with slight modifications. KB3.1 cells were grown as monolayer cultures in Dulbecco's modified Eagle's medium (DMEM) containing 10% fetal calf serum at 37 °C in 24-well multidishes. After three days, the confluent monolayers were washed twice with phosphate buffered saline (PBS), and the reaction mixture (450 µL Hanks' buffer at pH 7.2 containing 50 or 100 µM substrate ʟ-Leu-AMC, compounds dissolved in 50 µL DMSO) was added. After being incubated at 23 °C for 30 min, 1 mL cold 0.2 M glycine buffer at pH 10.5 was added. The amount of hydrolyzed 7-amino-4-methylcoumarin (AMC) was measured in a Tecan Infinite M200 PRO fluorescence spectrophotometer (excitation and emission at λ = 365 and 440 nm, respectively). Bestatin [12] and DMSO were used as positive and negative control, respectively.

## Supporting Information

File 1 HRESIMS data, NMR spectra of metabolites, media composition for incubation of microorganisms, and ITS sequences of the producing strain.
