# Supplementary material for: Skeletocutins M–Q: biologically active compounds from the fruiting bodies of the basidiomycete Skeletocutis sp. collected in Africa
Source: Beilstein J Org Chem. 2019 Nov 19;15:2782–9. doi: 10.3762/bjoc.15.270 (PMC6880814; doi:10.3762/bjoc.15.270)
Supplement: File 1 — HRESIMS data, NMR spectra of metabolites, media composition for incubation of microorganisms, and ITS sequences of the producing strain. [file Beilstein_J_Org_Chem-15-2782-s001.pdf]

BEILSTEIN JOURNAL OF ORGANIC CHEMISTRY

# Supporting Information

for

# Skeletocutins M–Q: biologically active compounds from the fruiting bodies of the basidiomycete *Skeletocutis* sp. collected in Africa

Tian Cheng, Clara Chepkirui, Cony Decock, Josphat C. Matasyoh and Marc Stadler

*Beilstein J. Org. Chem.* **2019,** *15,* 2782–2789. doi:10.3762/bjoc.15.270

# HRESIMS data, NMR spectra of metabolites, media composition for incubation of microorganisms, and ITS sequences of the producing strain

License and Terms: This is a supporting information file under the terms of the Creative Commons Attribution License (http://creativecommons.org/licenses/by/4.0). Please note that the reuse, redistribution and reproduction in particular requires that the authors and source are credited.
The license is subject to the *Beilstein Journal of Organic Chemistry* terms and conditions: (https://www.beilstein-journals.org/bjoc)

## Content


1 and 2D NMR data for skeletocutin M (**1**) ..................... S1

Figure S1: $^{1}H$ NMR spectrum of skeletocutin M (**1**) in acetone-$d_6$ (500 MHz) ..................... S1

Figure S2: $^{13}C$ NMR spectrum of skeletocutin M (**1**) in acetone-$d_6$ (125 MHz) ..................... S1

Figure S3: DEPT NMR spectrum of skeletocutin M (**1**) in acetone-$d_6$ (125 MHz) ..................... S2

Figure S4: $^{1}H$, $^{13}C$ HSQC spectrum of skeletocutin M (**1**) in acetone-$d_6$ (500 MHz, 125 MHz) ..................... S2

Figure S5: $^{1}H$, $^{13}C$ HMBC spectrum of skeletocutin M (**1**) in acetone-$d_6$ (500 MHz, 125 MHz) ..................... S3

Figure S6: $^{1}H$, $^{1}H$ COSY spectrum of skeletocutin M (**1**) in acetone-$d_6$ (500 MHz) ..................... S3

Figure S7: HRESIMS spectrum of skeletocutin M (**1**) ..................... S4

1 and 2D NMR data for skeletocutin N (**2**) ..................... S4

Figure S8: $^{1}H$ NMR spectrum of skeletocutin N (**2**) in CDCl3 (500 MHz) ..................... S4

Figure S9: $^{13}C$ NMR spectrum of skeletocutin N (**2**) in $CDCl_3$ (125 MHz) ..................... S5

Figure S10: DEPT NMR spectrum of skeletocutin N (**2**) in $CDCl_3$ (125 MHz) ..................... S5

Figure S11: $^{1}H$, $^{13}C$ HSQC spectrum of skeletocutin N (**2**) in $CDCl_3$ (500 MHz, 125 MHz) ..................... S6

Figure S12: $^{1}H$, $^{13}C$ HMBC spectrum of skeletocutin N (**2**) in $CDCl_3$ (500 MHz, 125 MHz) ..................... S6

Figure S13: $^{1}H$, $^{1}H$ COSY spectrum of skeletocutin N (**2**) $CDCl_3$ (500 MHz) ..................... S7

Figure S14: HRESIMS spectrum of skeletocutin N (**2**) ..................... S7

1 and 2D NMR data for skeletocutin O (**3**) ..................... S8

Figure S15: $^{1}H$ NMR spectrum of skeletocutin O (**3**) in $CDCl_3$ (500 MHz) ..................... S8

Figure S16: Expanded $^{1}H$ NMR spectrum of skeletocutin O (**3**) in $CDCl_3$ (500 MHz) ..................... S8

Figure S17: $^{13}C$ NMR spectrum of skeletocutin O (**3**) in $CDCl_3$ (125 MHz) ..................... S9

Figure S18: DEPT NMR spectrum of skeletocutin O (**3**) in $CDCl_3$ 125 MHz) ..................... S9

Figure S19: $^{1}H$, $^{13}C$ HSQC spectrum of skeletocutin O (**3**) in $CDCl_3$ (500 MHz, 125 MHz) ..................... S10

Figure S20: $^{1}H$, $^{13}C$ HMBC spectrum of skeletocutin O (**3**) in $CDCl_3$ (500 MHz, 125 MHz) ..................... S10

Figure S21: $^{1}H$, $^{1}H$ COSY spectrum of skeletocutin O (**3**) $CDCl_3$ (500 MHz) ..................... S11

Figure S22: $^{1}H$, $^{1}H$ ROESY spectrum of skeletocutin O (**3**) CDCl3 (500 MHz) ..................... S11

Figure S23: HRESIMS spectrum of skeletocutin O (**3**) ..................... S12

1 and 2D NMR data for skeletocutin P (**4**) ..................... S12

Figure S24: $^{1}H$ NMR spectrum of skeletocutin P (**4**) in DMSO (500 MHz) ...................... S12
Figure S25: $^{13}C$ NMR spectrum of skeletocutin P (**4**) in DMSO (125 MHz) ...................... S13
Figure S26: DEPT NMR spectrum of skeletocutin P (**4**) in DMSO (125 MHz) ................ S13
Figure S27: $^{1}H$, $^{13}C$ HSQC spectrum of skeletocutin P (**4**) in DMSO (500 MHz, 125 MHz) ........................................................................................ S14
Figure S28: $^{1}H$, $^{13}C$ HMBC spectrum of skeletocutin P (**4**) in DMSO (500 MHz, 125 MHz) ........................................................................................ S14
Figure S29: $^{1}H$, $^{1}H$ COSY spectrum of skeletocutin O (**4**) in DMSO (500 MHz) .............. S15
Figure S30: $^{1}H$, $^{1}H$ ROESY spectrum of skeletocutin O (**4**) in DMSO (500 MHz) ............ S15
Figure S31: HRESIMS spectrum of skeletocutin P (**4**) .................................................. S16
1 and 2D NMR data for skeletocutin Q (**5**) .......................................................... S17
Figure S32: $^{1}H$ NMR spectrum of skeletocutin Q (**5**) in DMSO (500 MHz) ..................... S17
Figure S33: $^{13}C$ NMR spectrum of skeletocutin Q (**5**) in DMSO (125 MHz) ..................... S17
Figure S34: DEPT NMR spectrum of skeletocutin Q (**5**) in DMSO (125 MHz) ................ S18
Figure S35: $^{1}H$, $^{13}C$ HSQC spectrum of skeletocutin Q (**5**) in DMSO (500 MHz, 125 MHz) ........................................................................................ S18
Figure S36: $^{1}H$, $^{13}C$ HMBC spectrum of skeletocutin Q (**5**) in DMSO (500 MHz, 125 MHz) ........................................................................................ S19
Figure S37: $^{1}H$, $^{1}H$ COSY spectrum of skeletocutin Q (**5**) in DMSO (500 MHz) .............. S19
Figure S38: $^{1}H$, $^{1}H$ ROESY spectrum of skeletocutin Q (**5**) in DMSO (500 MHz) ............ S20
Figure S39: HRESIMS spectrum of skeletocutin Q (**5**) .................................................. S20
Media ........................................................................................................ S21
ITS sequence ............................................................................................ S21
Biofilm Inhibition ....................................................................................... S21

## 1 and 2D NMR data for skeletocutin M (1)

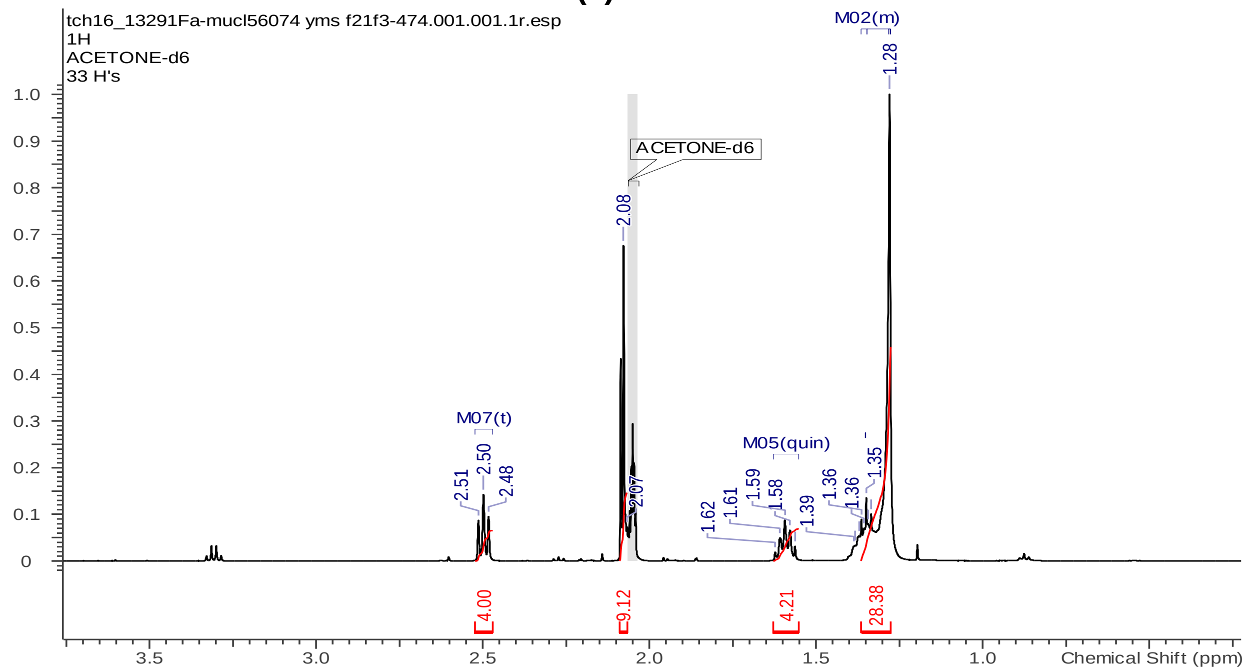


Figure S1: $^{1}$H NMR spectrum of skeletocutin M (**1**) in acetone-$d_6$ (500 MHz)

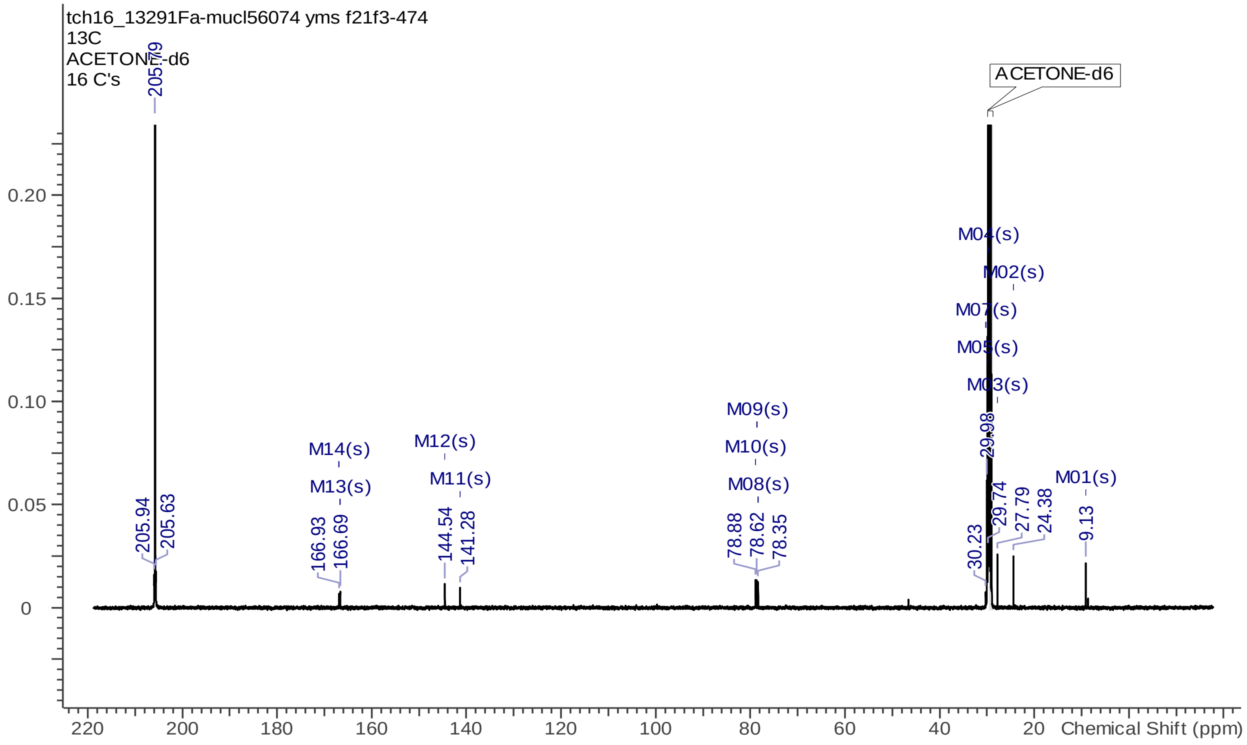


Figure S2: $^{13}$C NMR spectrum of skeletocutin M (**1**) in acetone-$d_6$ (125 MHz)

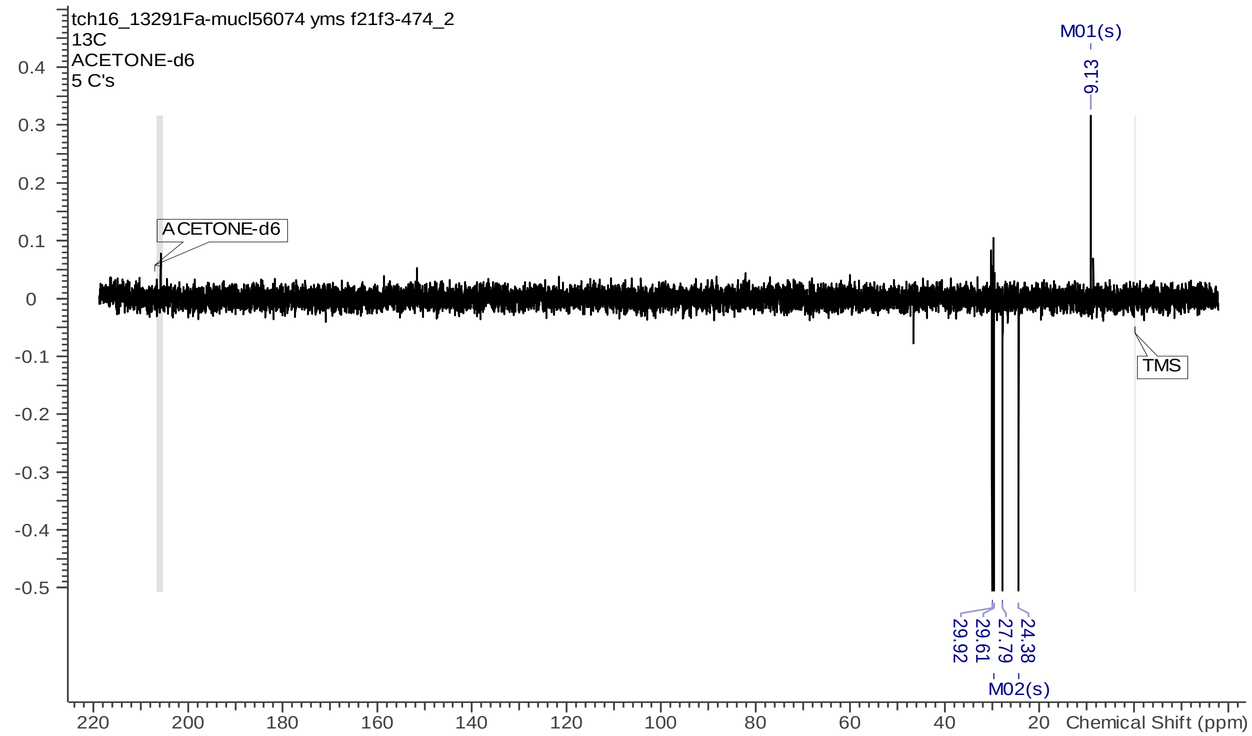


Figure S3: DEPT NMR spectrum of skeletocutin M (**1**) in acetone-$d_6$ (125 MHz)

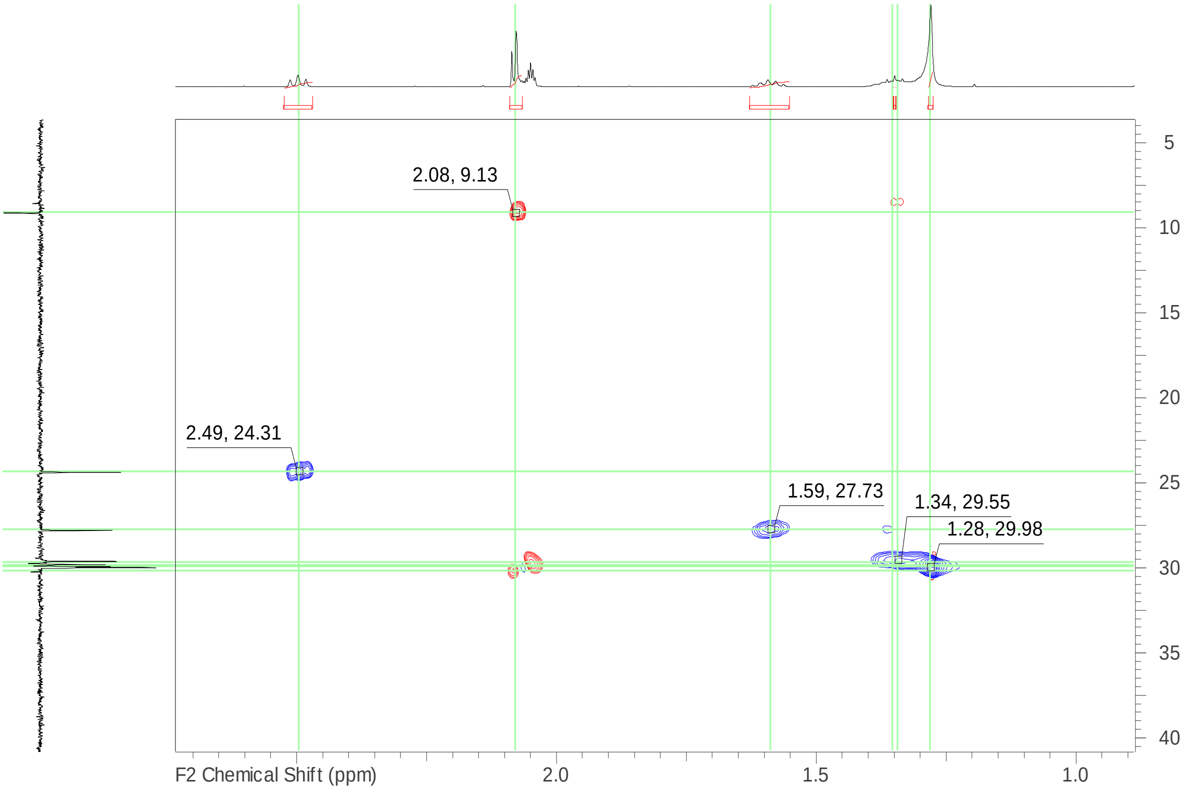


Figure S4: $^{1}H$, $^{13}C$ HSQC spectrum of skeletocutin M (**1**) in acetone-$d_6$ (500 MHz, 125 MHz)

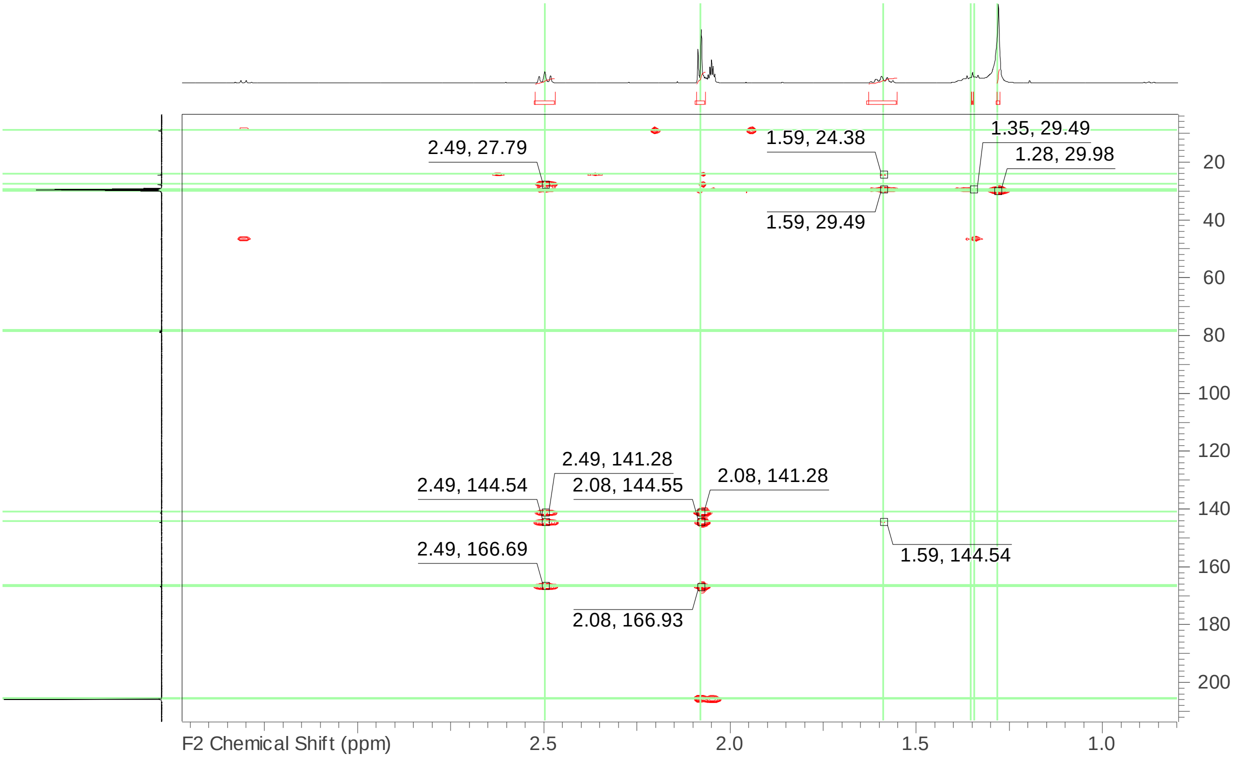


Figure S5: $^{1}$H, $^{13}$C HMBC spectrum of skeletocutin M (**1**) in acetone-$d_6$ (500 MHz, 125MHz)

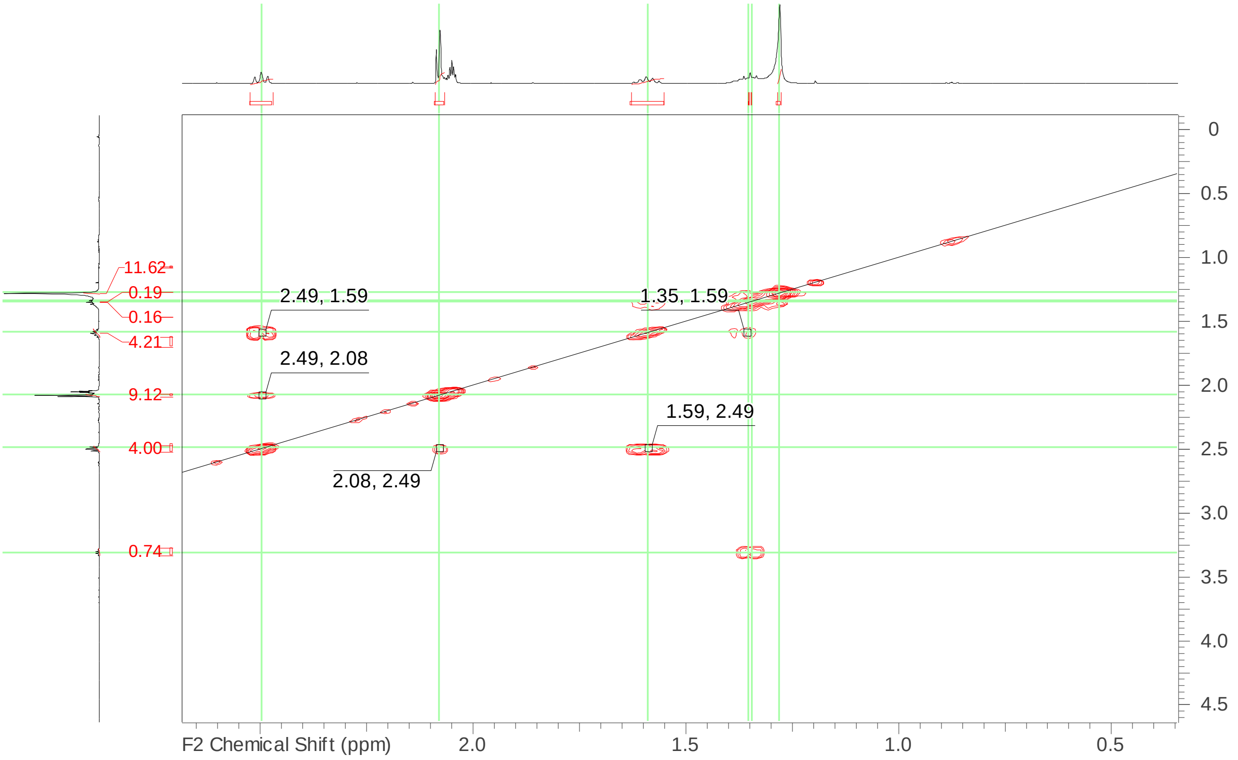


Figure S6: $^{1}$H, $^{1}$H COSY spectrum of skeletocutin M (**1**) in acetone-$d_6$ (500 MHz)

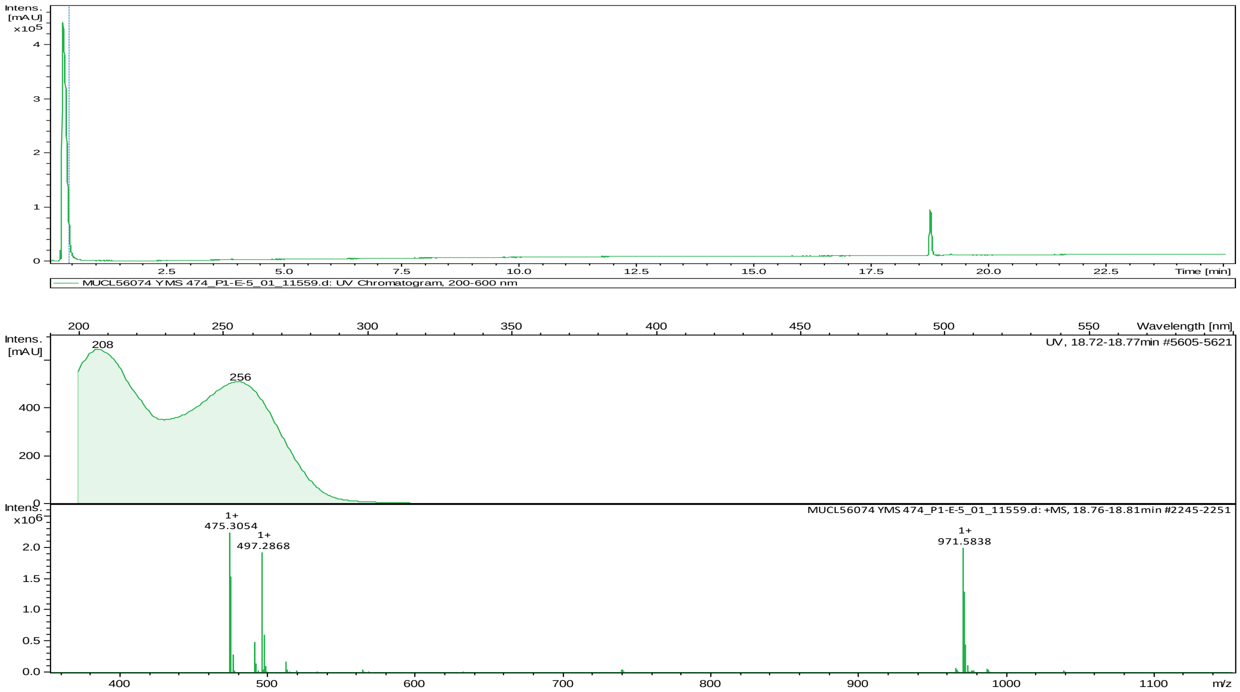

Figure S7: HRESIMS spectrum of skeletocutin M (**1**)

## 1 and 2D NMR data for skeletocutin N (2)

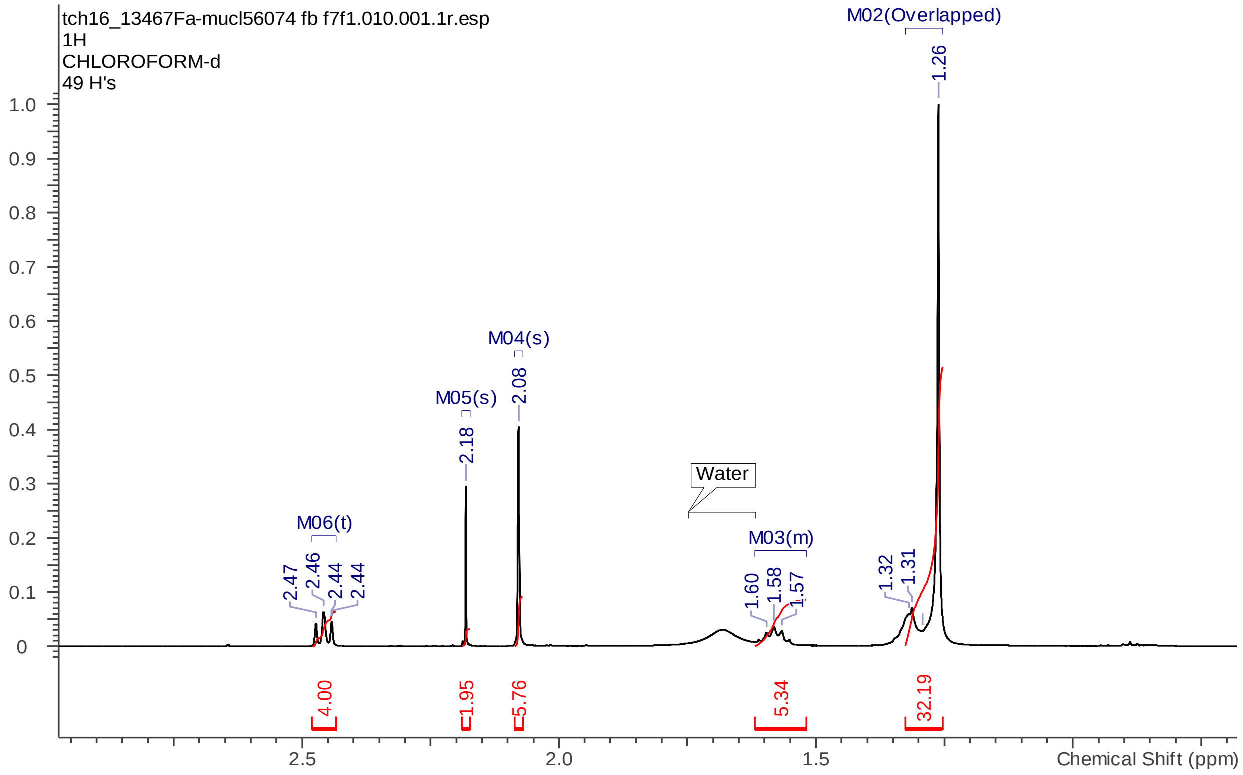

Figure S8: $^{1}$H NMR spectrum of skeletocutin N (**2**) in $CDCl_3$ (500 MHz)

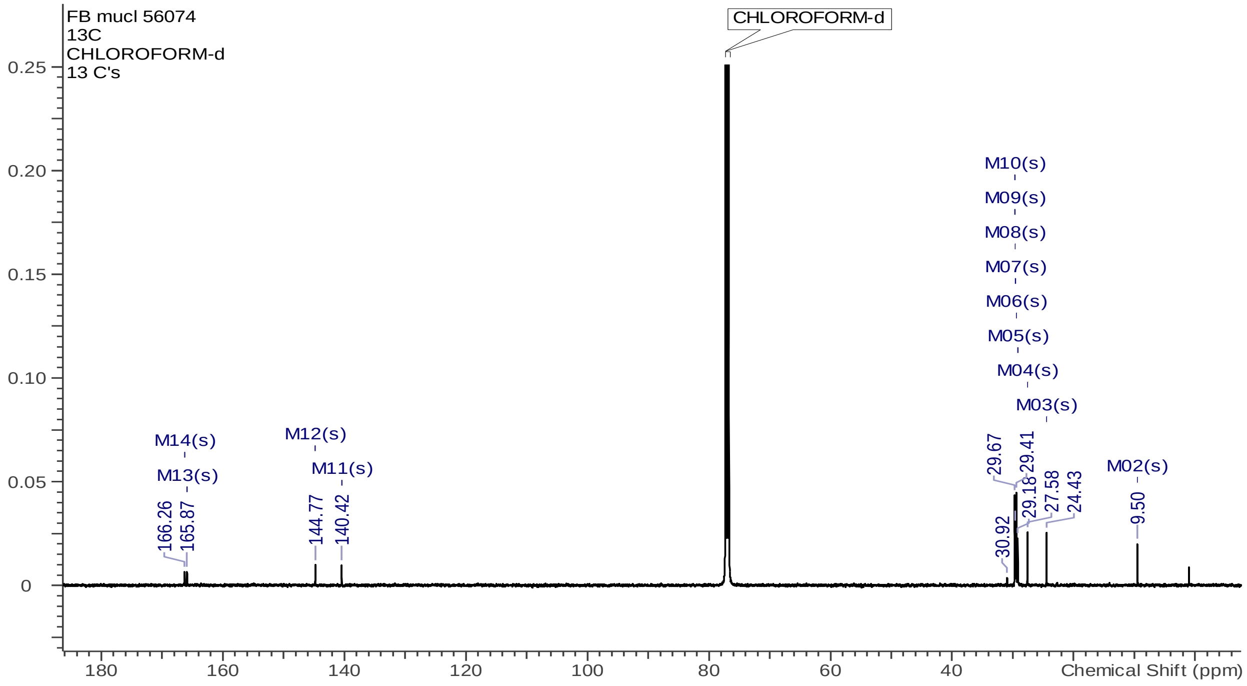


Figure S9: $^{13}$C NMR spectrum of skeletocutin N (**2**) in $CDCl_3$ (125 MHz)

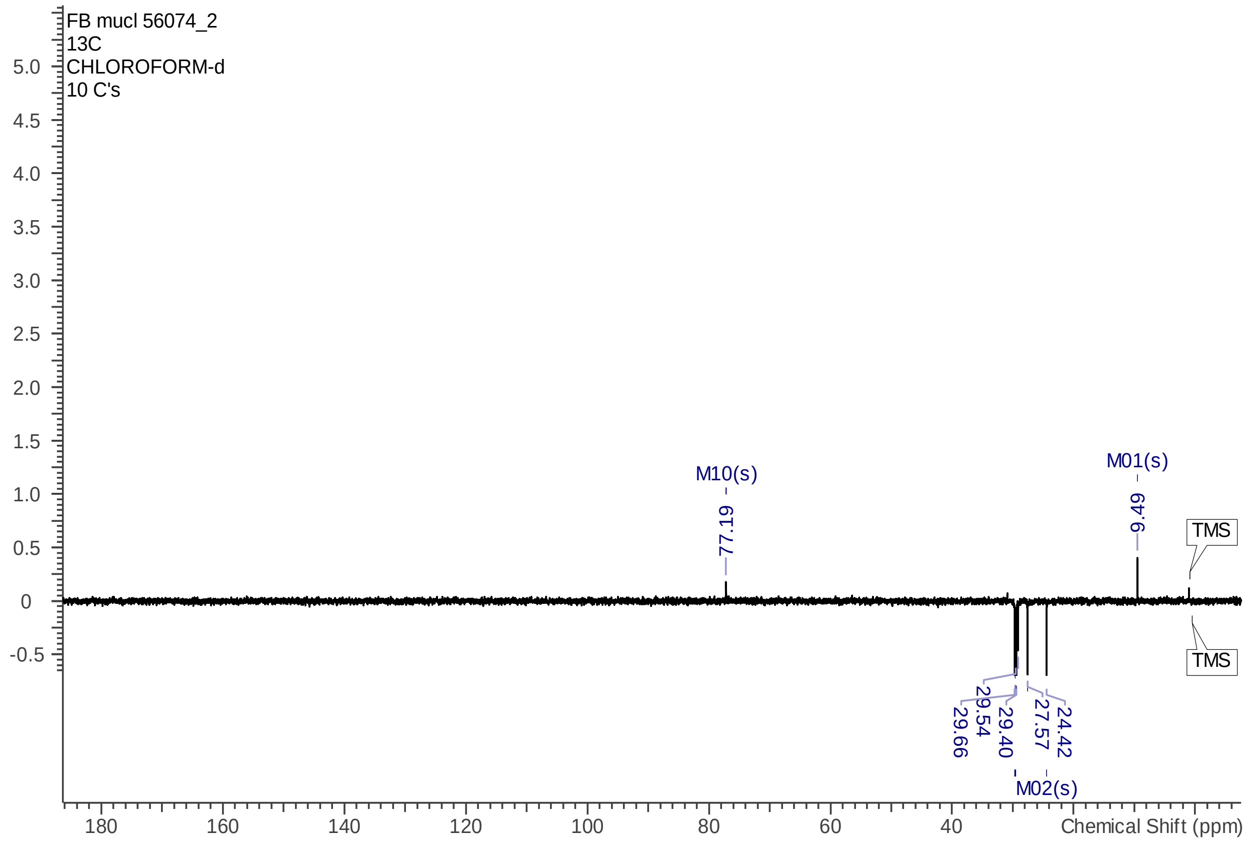


Figure S10: DEPT NMR spectrum of skeletocutin N (**2**) in $CDCl_3$ (125 MHz)

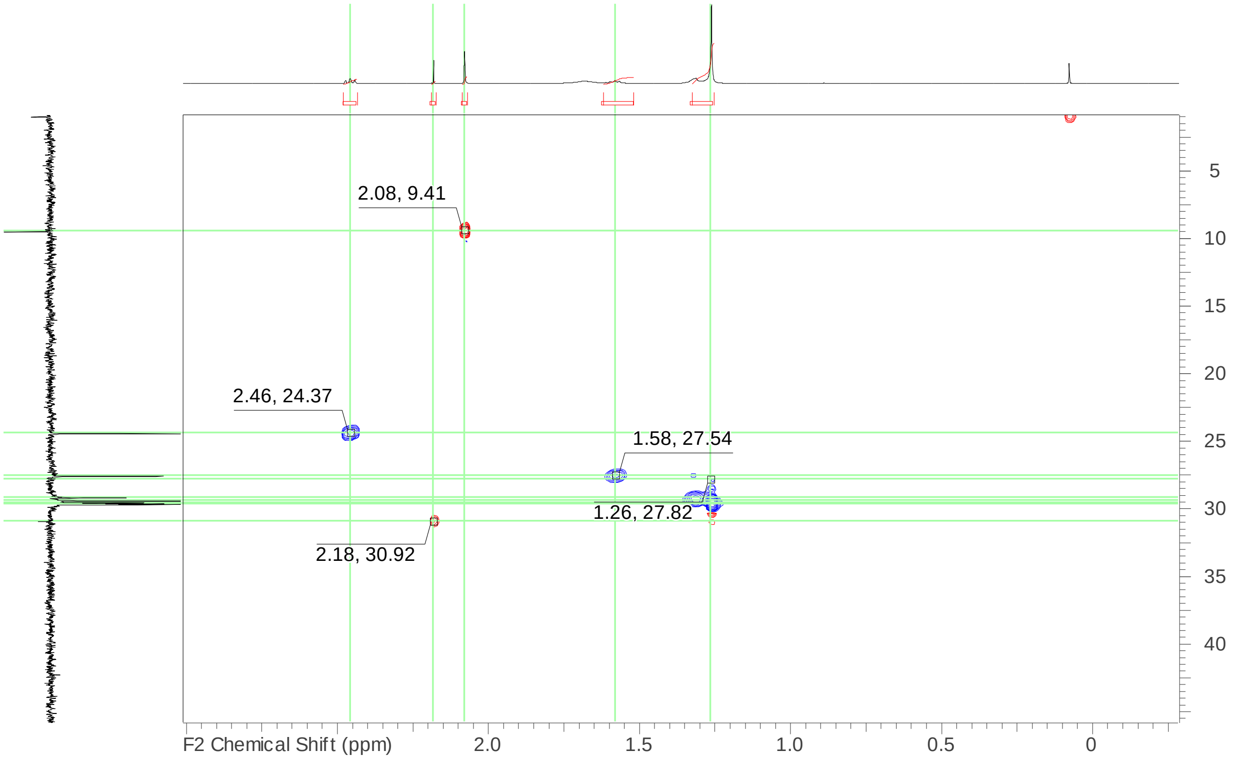


Figure S11: $^{1}$H, $^{13}$C HSQC spectrum of skeletocutin N (**2**) in $CDCl_3$ (500 MHz, 125 MHz)

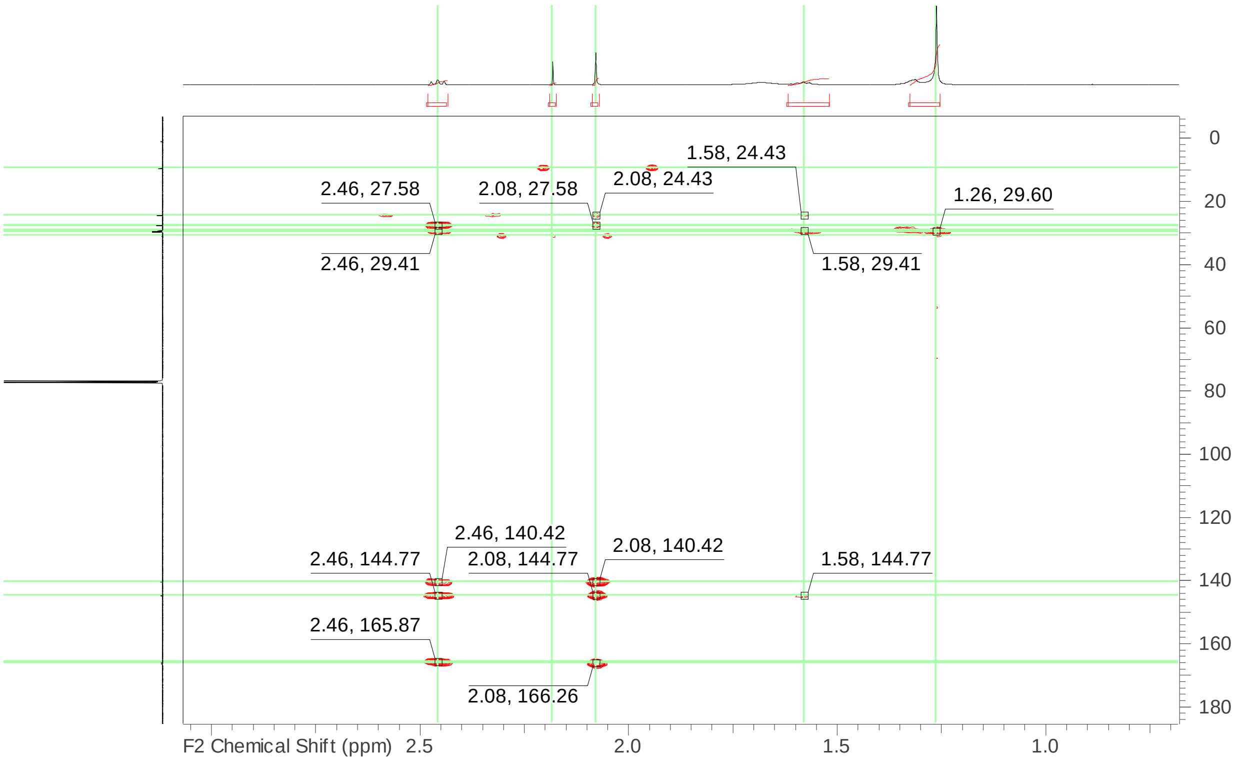


Figure S12: $^{1}$H, $^{13}$C HMBC spectrum of skeletocutin N (**2**) in $CDCl_3$ (500 MHz, 125 MHz)

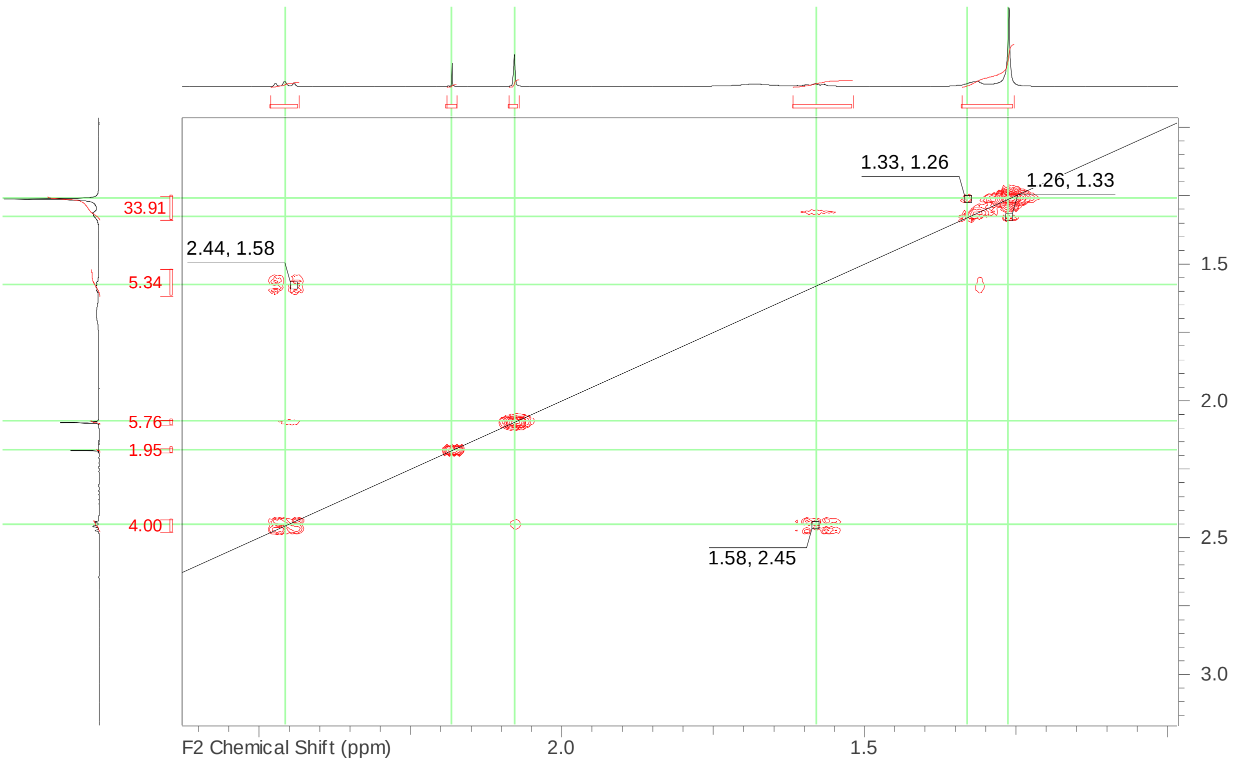


Figure S13: $^{1}H$, $^{1}H$ COSY spectrum of skeletocutin N (**2**) $CDCl_3$ (500 MHz)

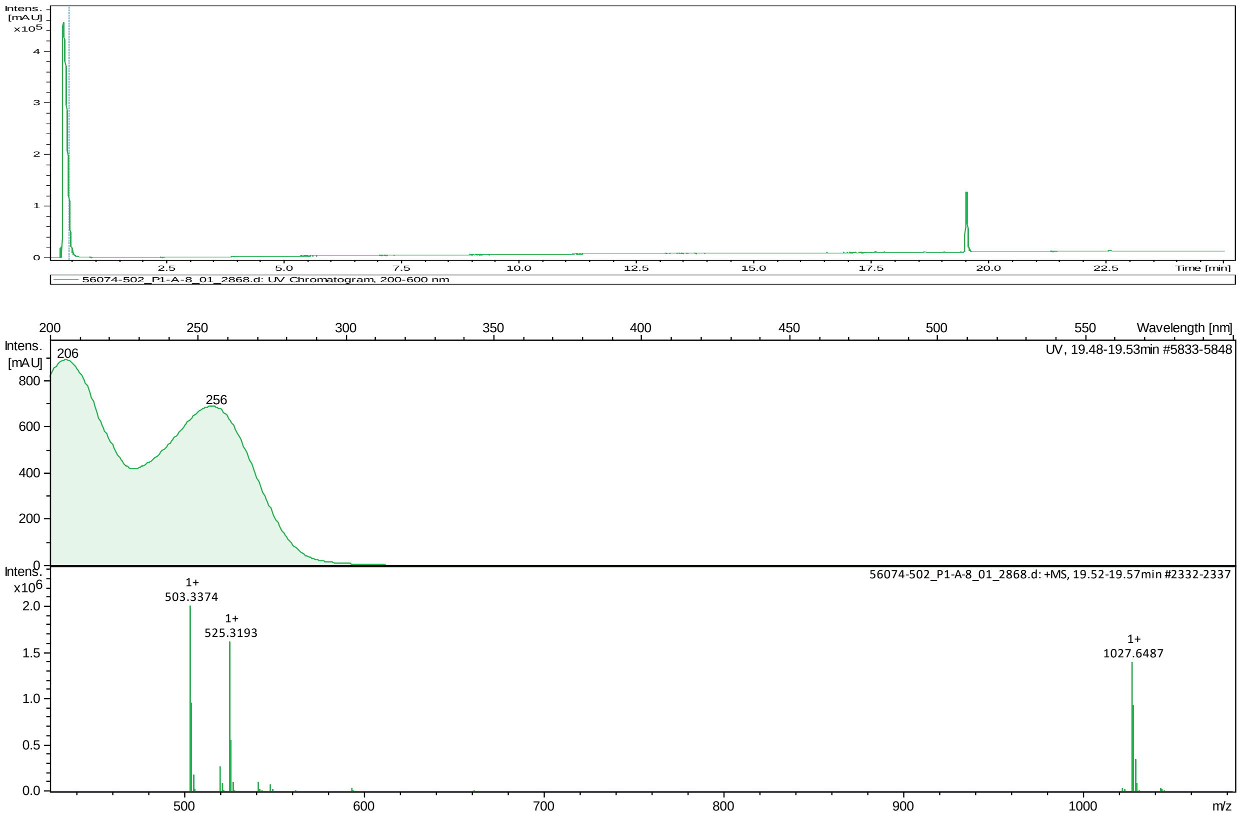


Figure S14: HRESIMS spectrum of skeletocutin N (**2**)

## 1 and 2D NMR data for skeletocutin O (3)

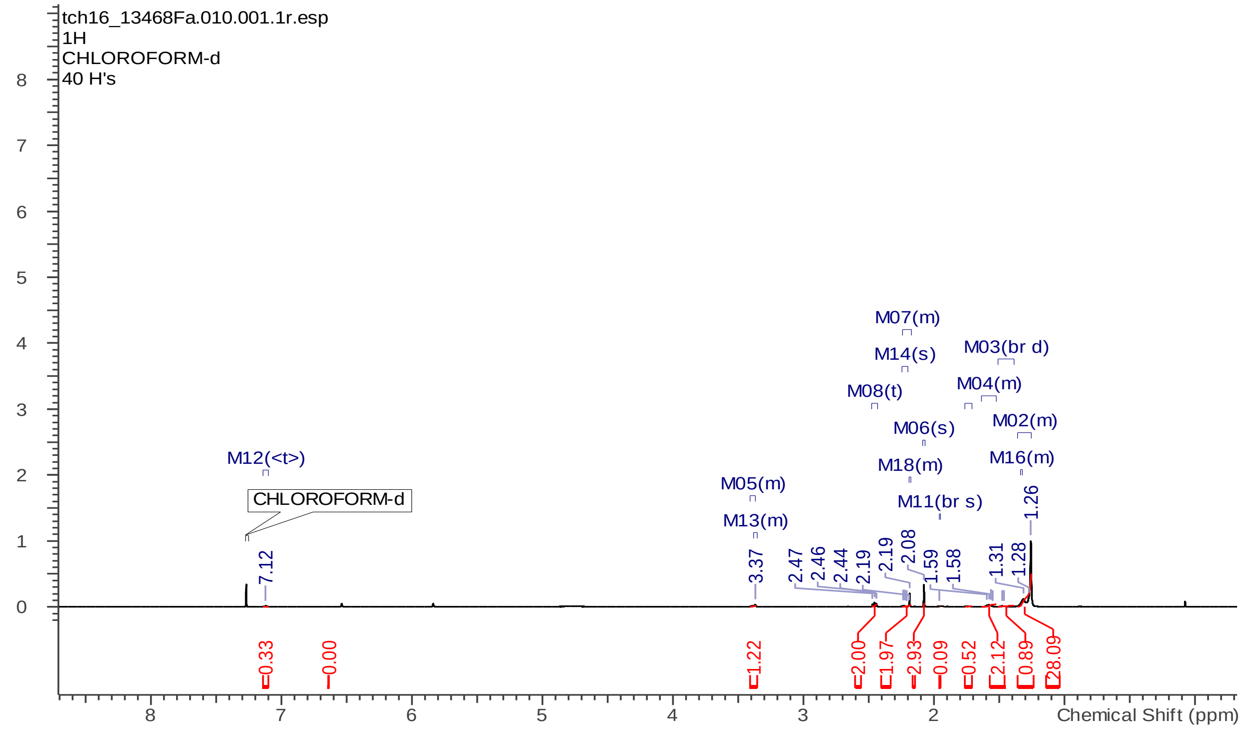


Figure S15: $^{1}$H NMR spectrum of skeletocutin O (3) in $CDCl_3$ (500 MHz)

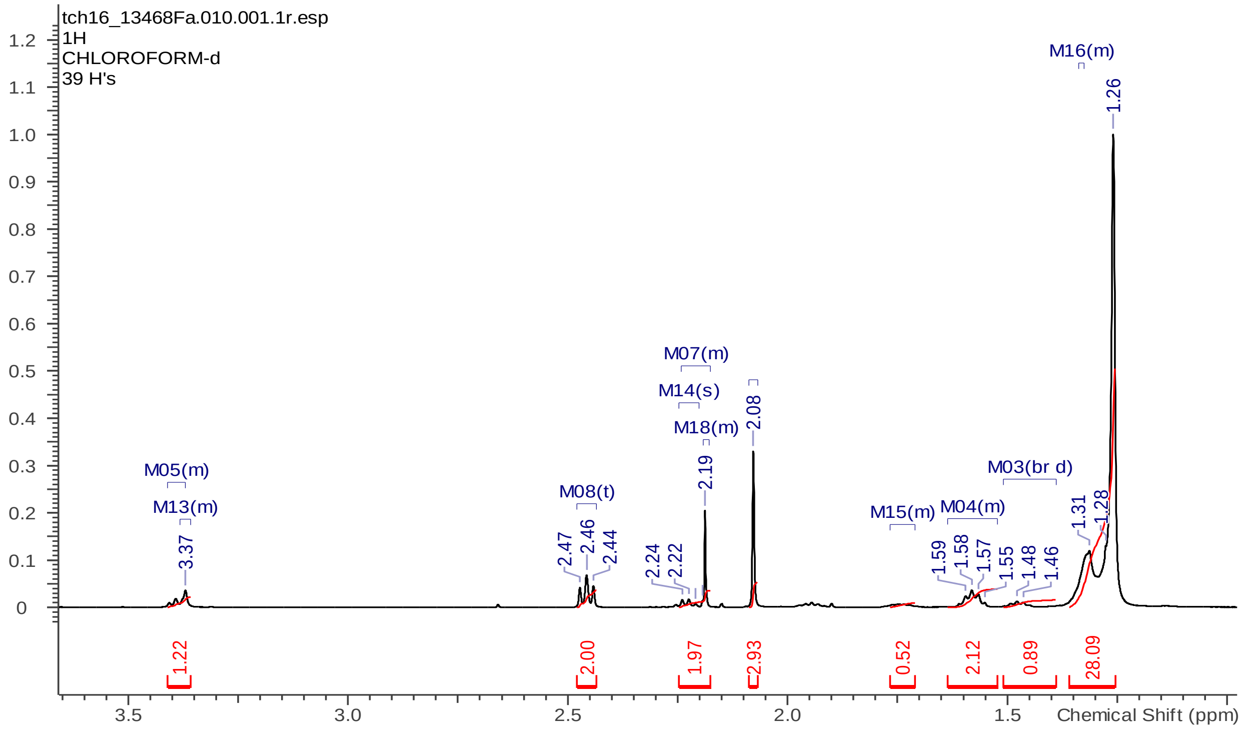


Figure S16: Expanded $^{1}$H NMR spectrum of skeletocutin O (**3**) in $CDCl_3$ (500 MHz)

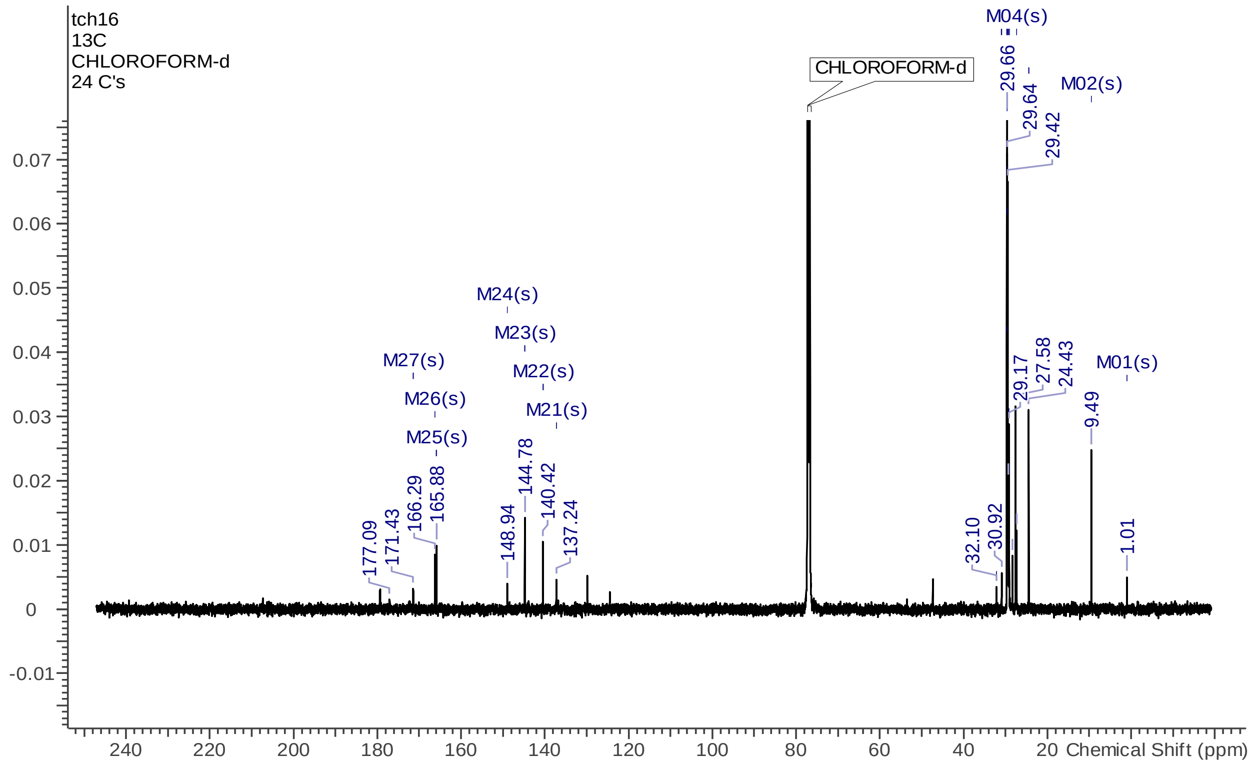


Figure S17: $^{13}C$ NMR spectrum of skeletocutin O (**3**) in $CDCl_3$ (125 MHz)

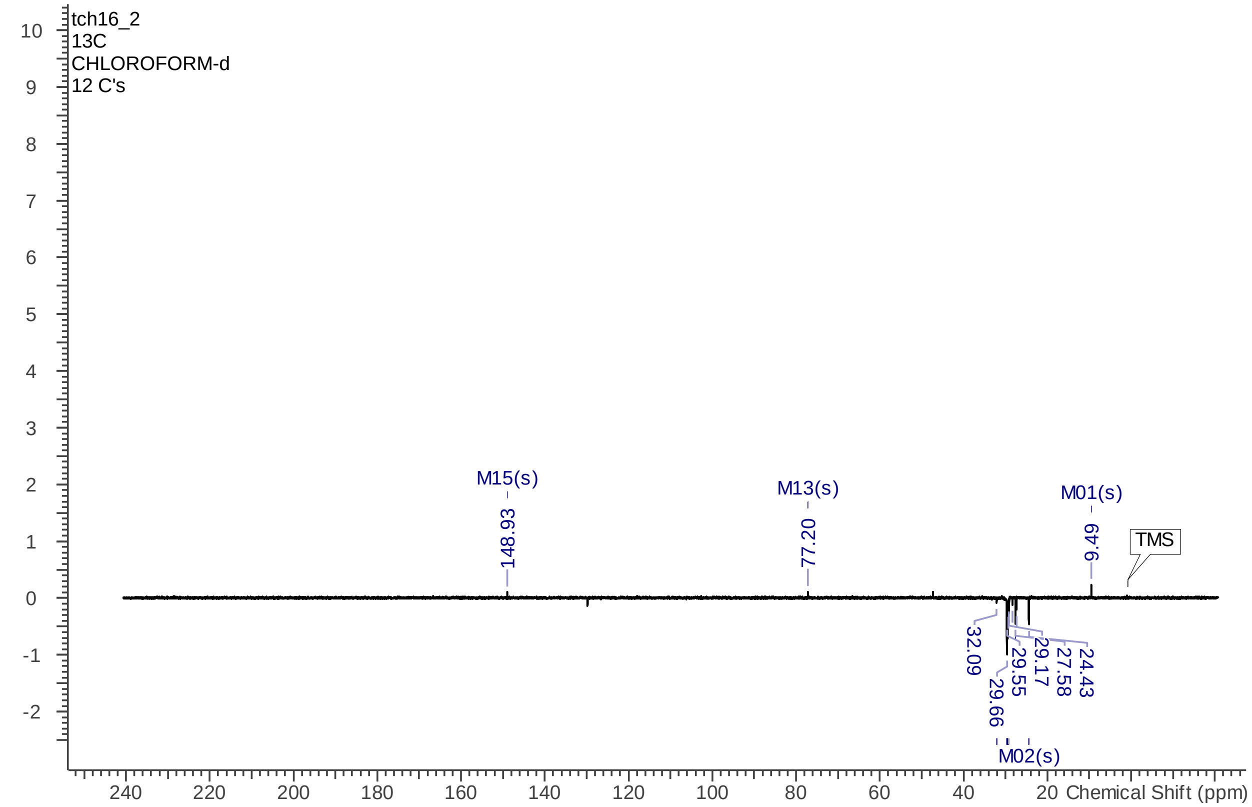


Figure S18: DEPT NMR spectrum of skeletocutin O (**3**) in $CDCl_3$ 125 MHz)

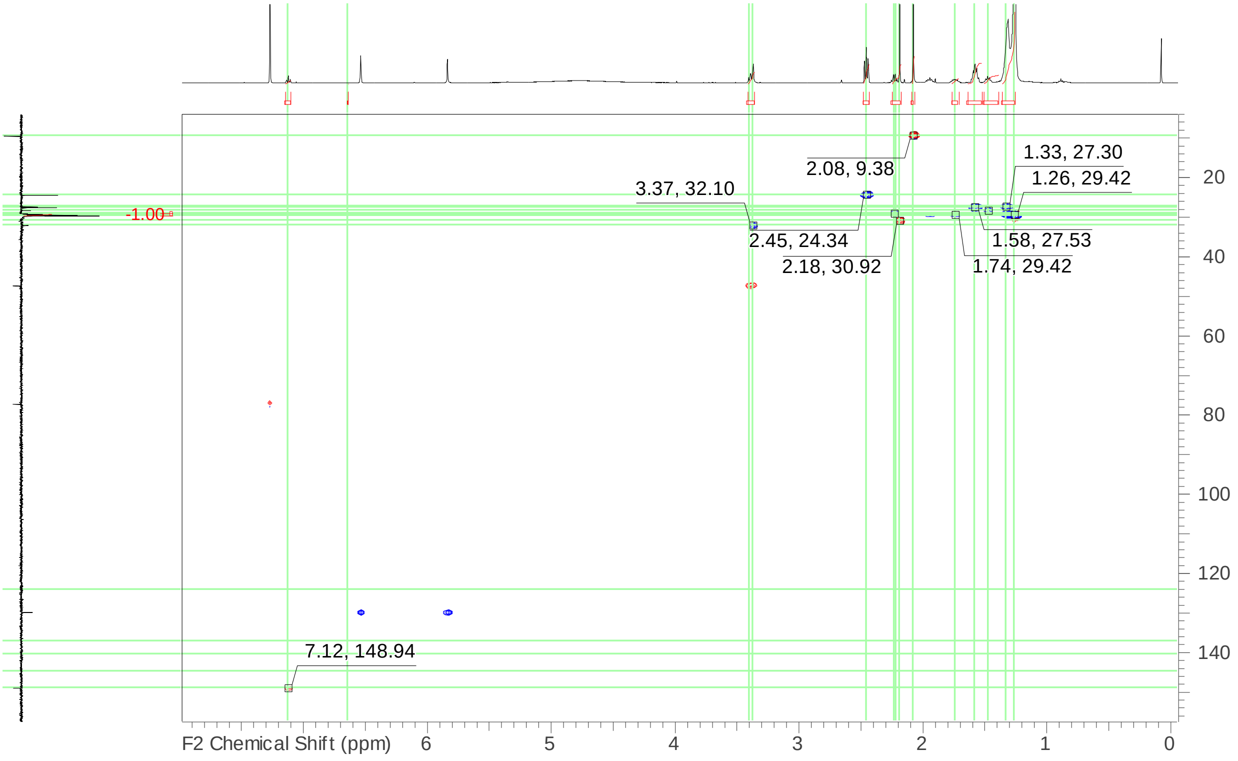


Figure S19: $^{1}H$, $^{13}C$ HSQC spectrum of skeletocutin O (**3**) in $CDCl_3$ (500 MHz, 125 MHz)

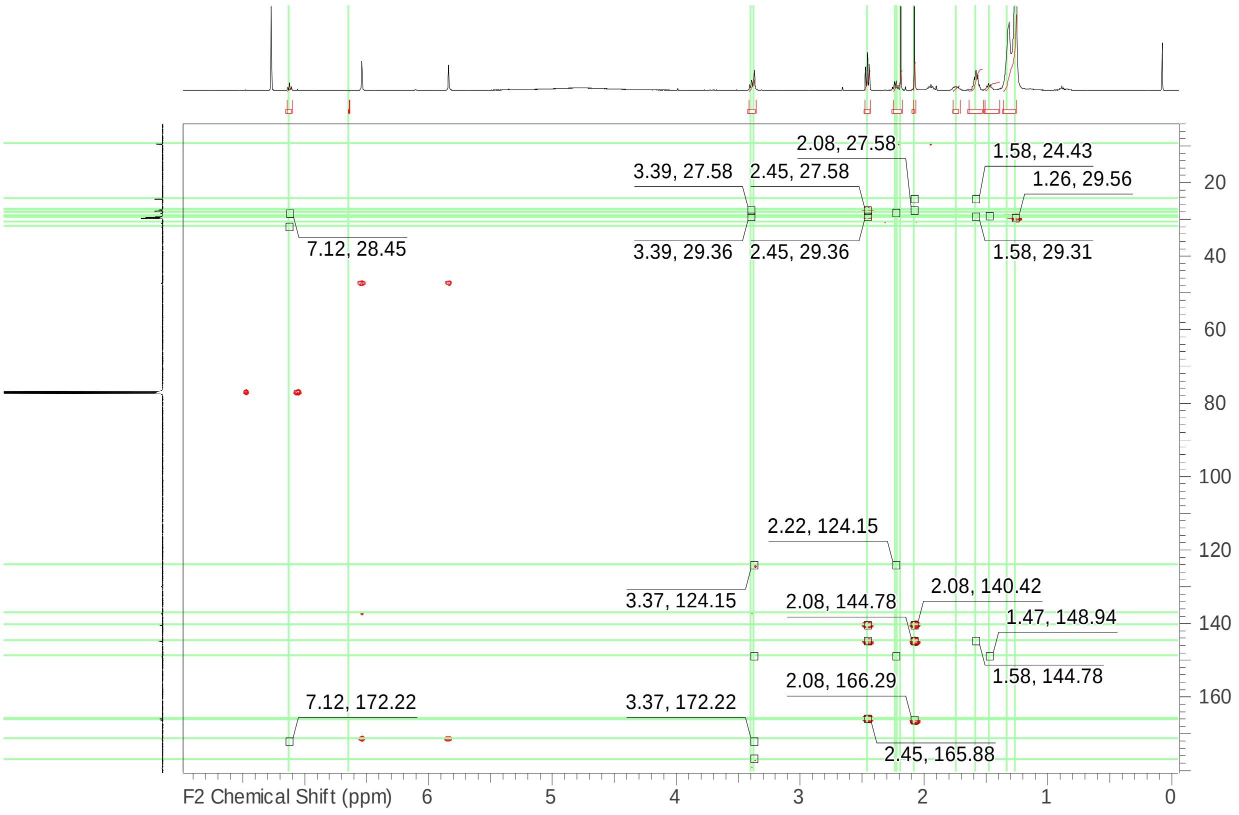


Figure S20: $^{1}H$, $^{13}C$ HMBC spectrum of skeletocutin O (**3**) in $CDCl_3$ (500 MHz, 125 MHz)

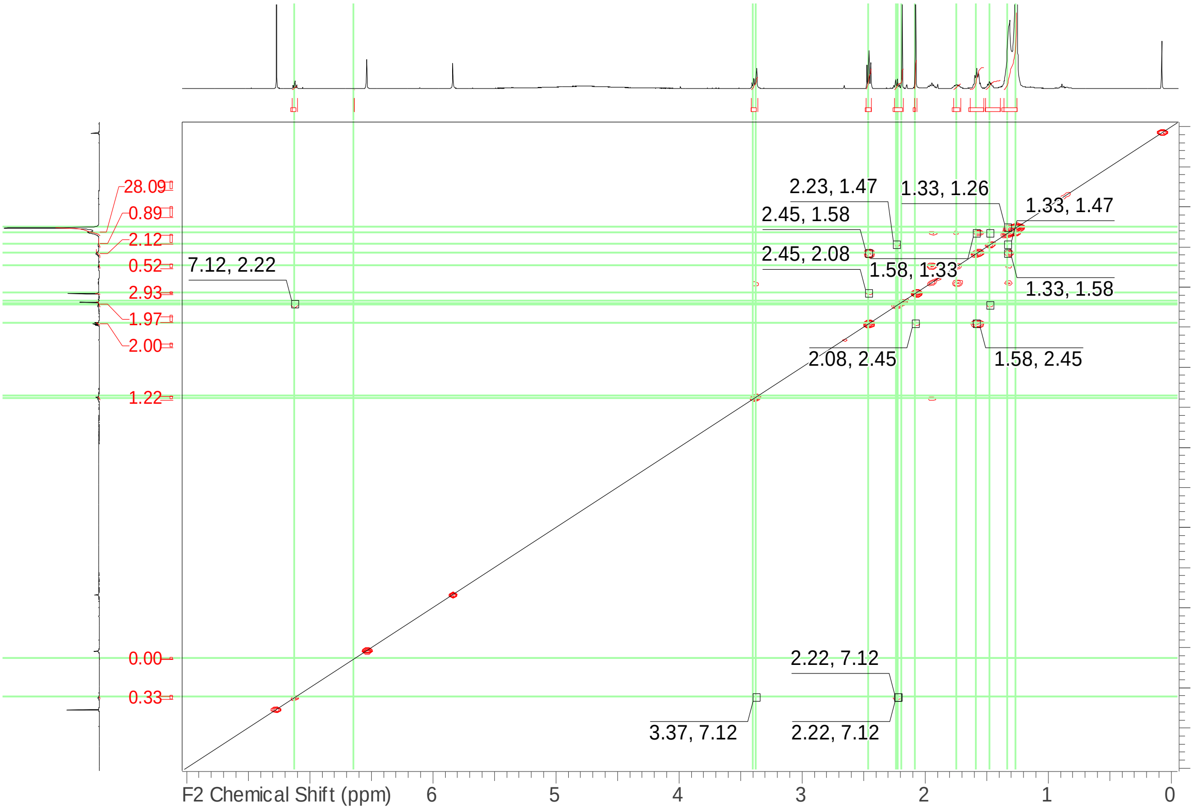


Figure S21: $^{1}H$, $^{1}H$ COSY spectrum of skeletocutin O (**3**) $CDCl_3$ (500 MHz)

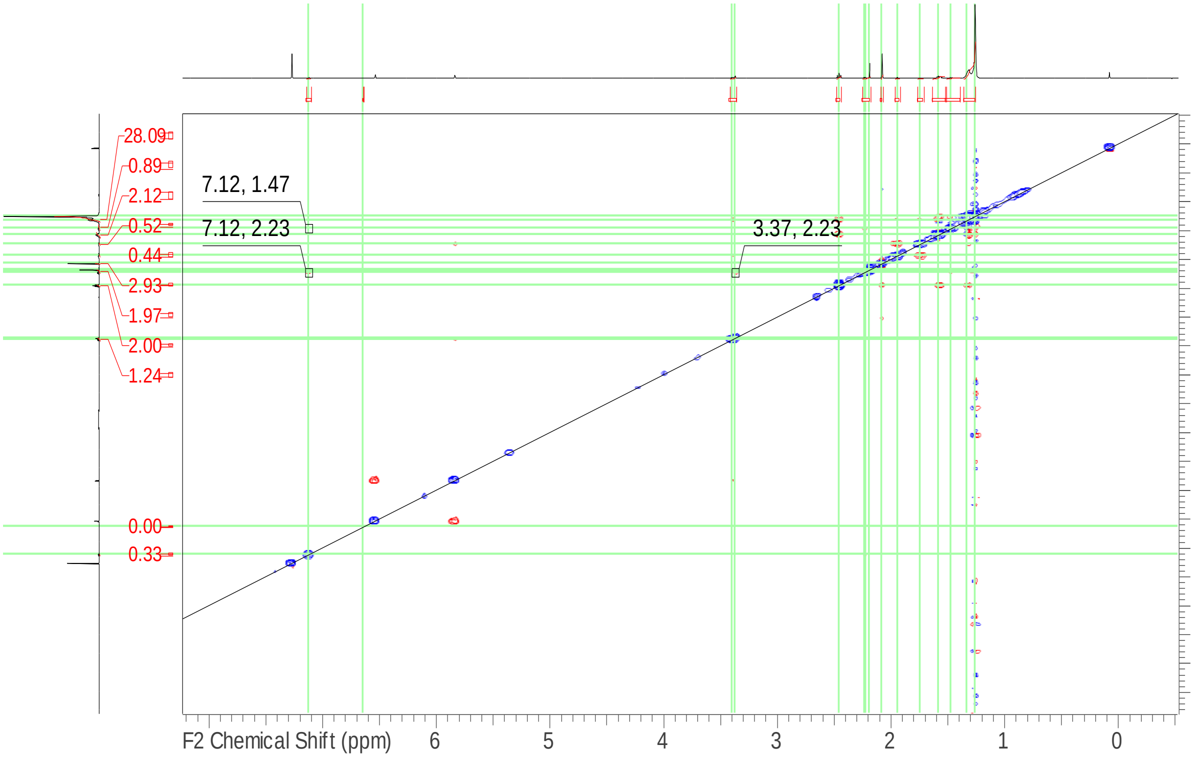


Figure S22: $^{1}H$, $^{1}H$ ROESY spectrum of skeletocutin O (**3**) $CDCl_3$ (500 MHz)

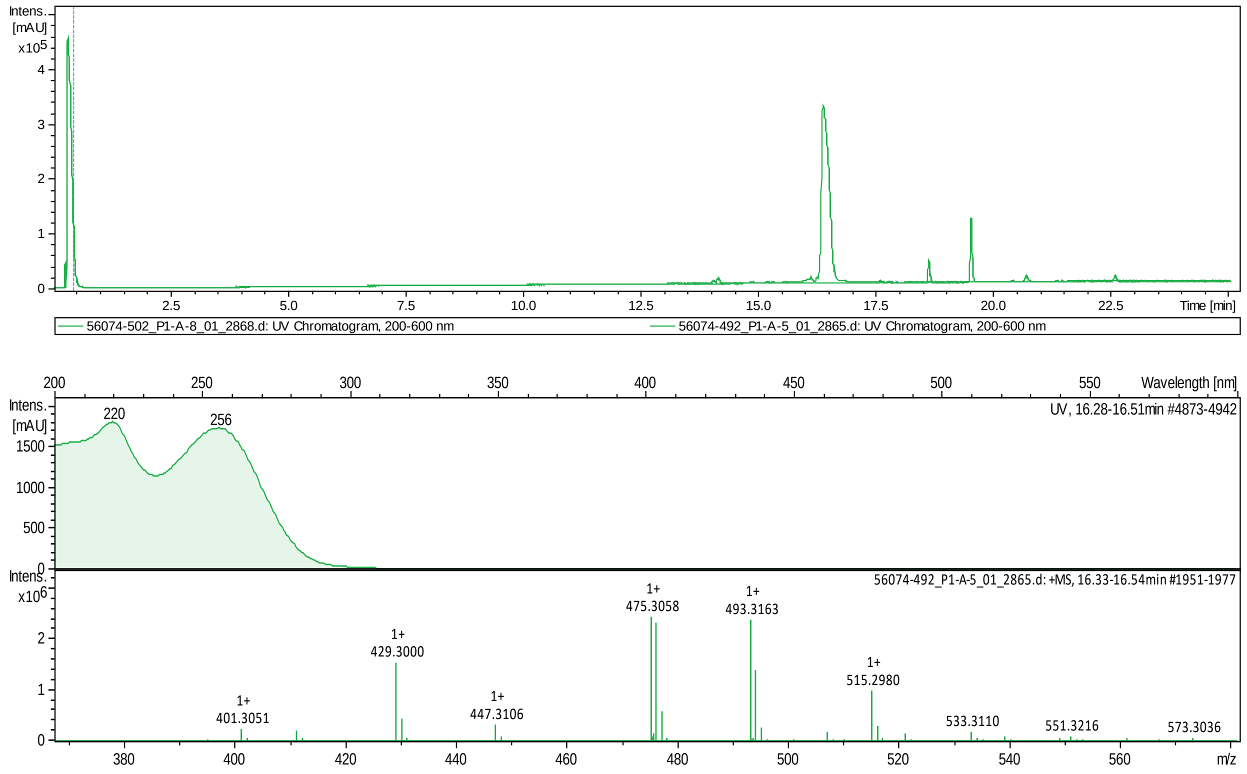


Figure S23: HRESIMS spectrum of skeletocutin O (**3**)

## 1 and 2D NMR data for skeletocutin P (4)

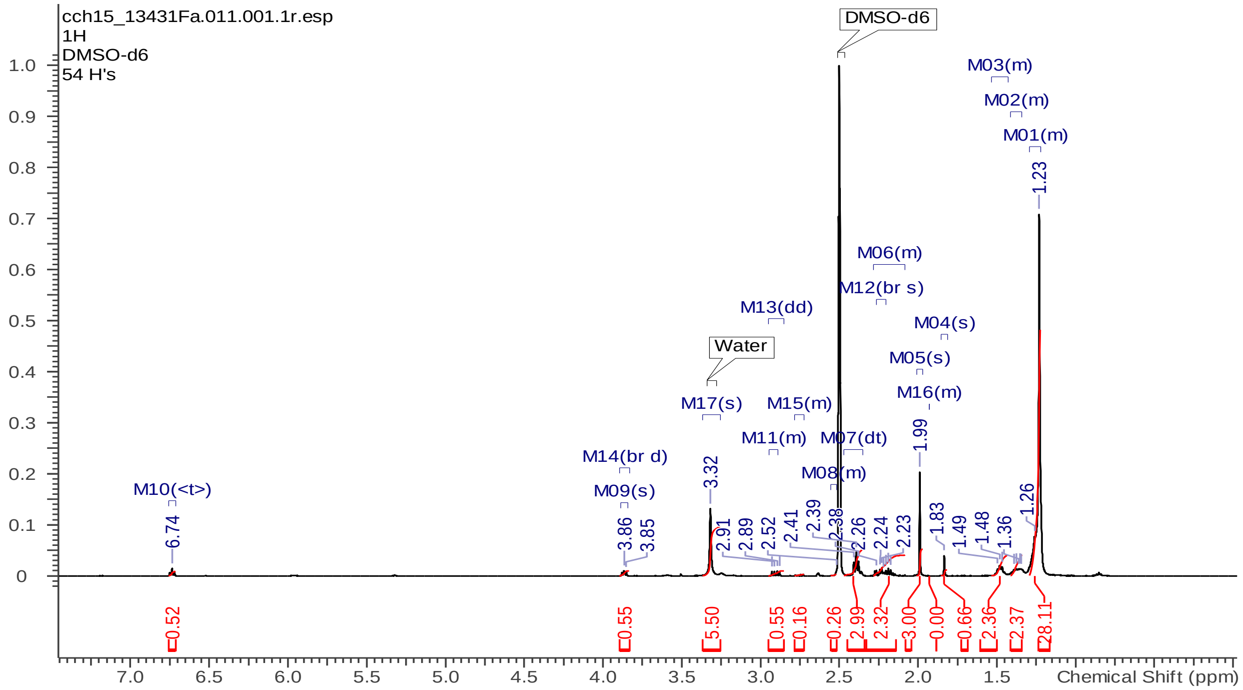


Figure S24: $^1$H NMR spectrum of skeletocutin P (**4**) in DMSO (500 MHz)

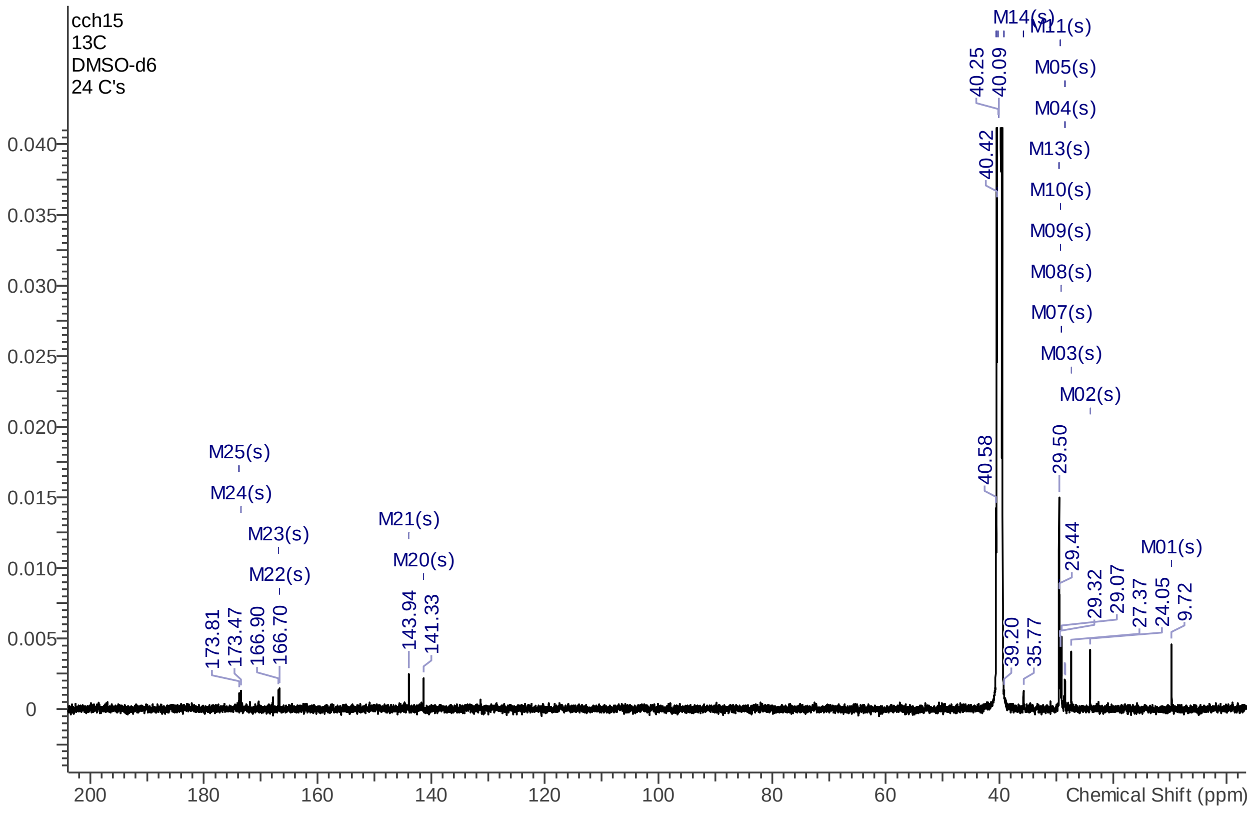


Figure S25: $^{13}$C NMR spectrum of skeletocutin P (**4**) in DMSO (125 MHz)

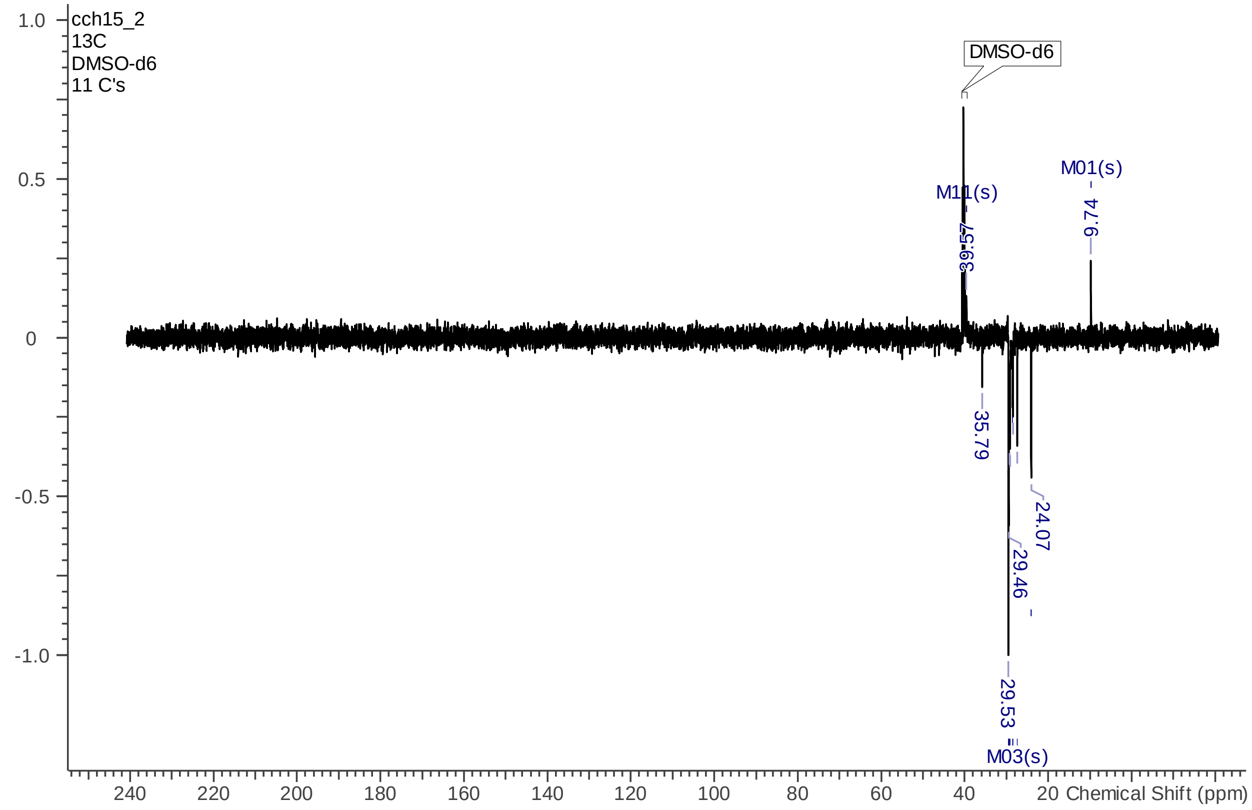


Figure S26: DEPT NMR spectrum of skeletocutin P (4) in DMSO (125 MHz)

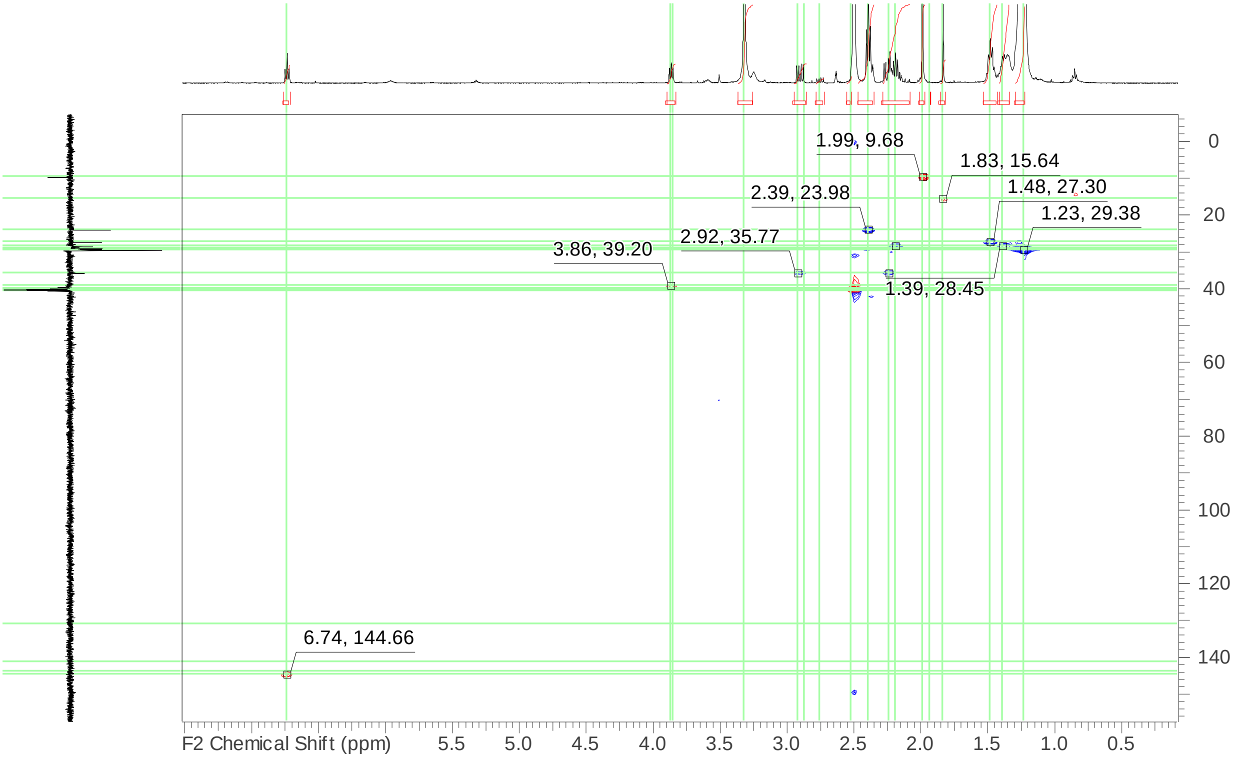


Figure S27: $^{1}$H, $^{13}$C HSQC spectrum of skeletocutin P (**4**) in DMSO (500 MHz, 125 MHz)

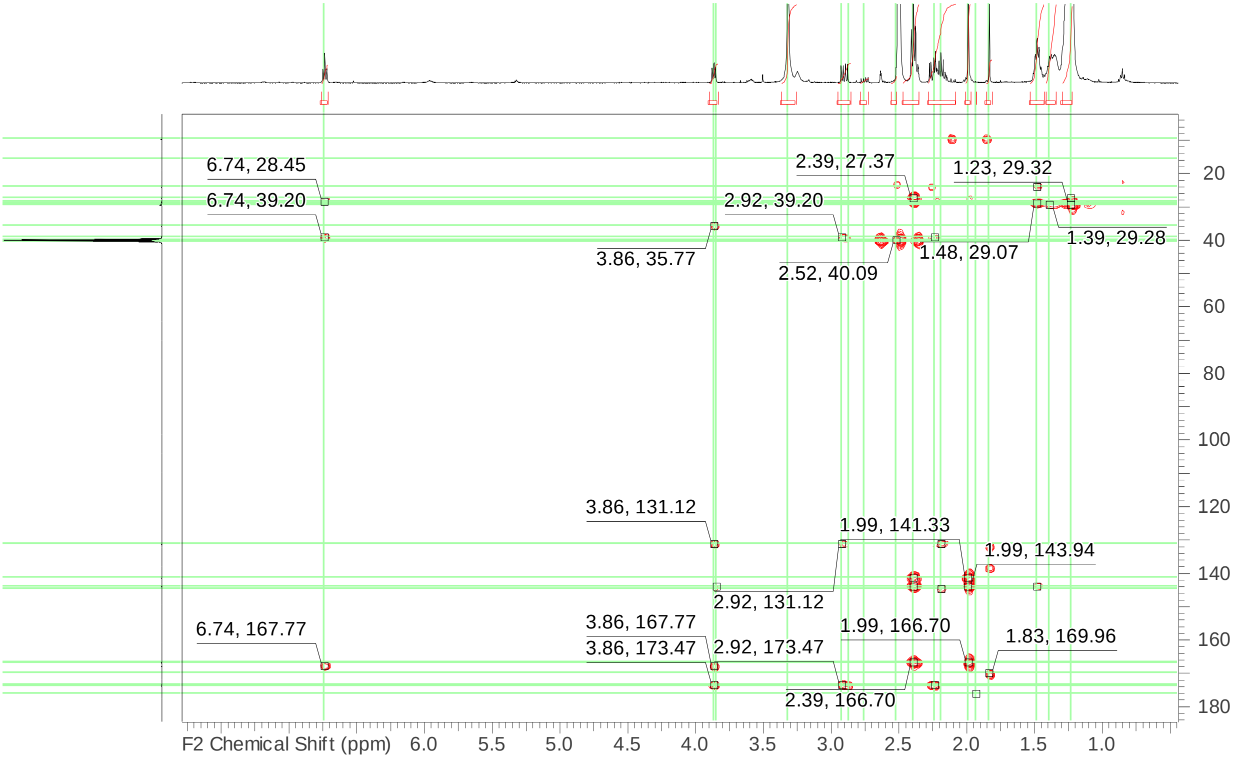


Figure S28: $^{1}$H, $^{13}$C HMBC spectrum of skeletocutin P (**4**) in DMSO (500 MHz, 125 MHz)

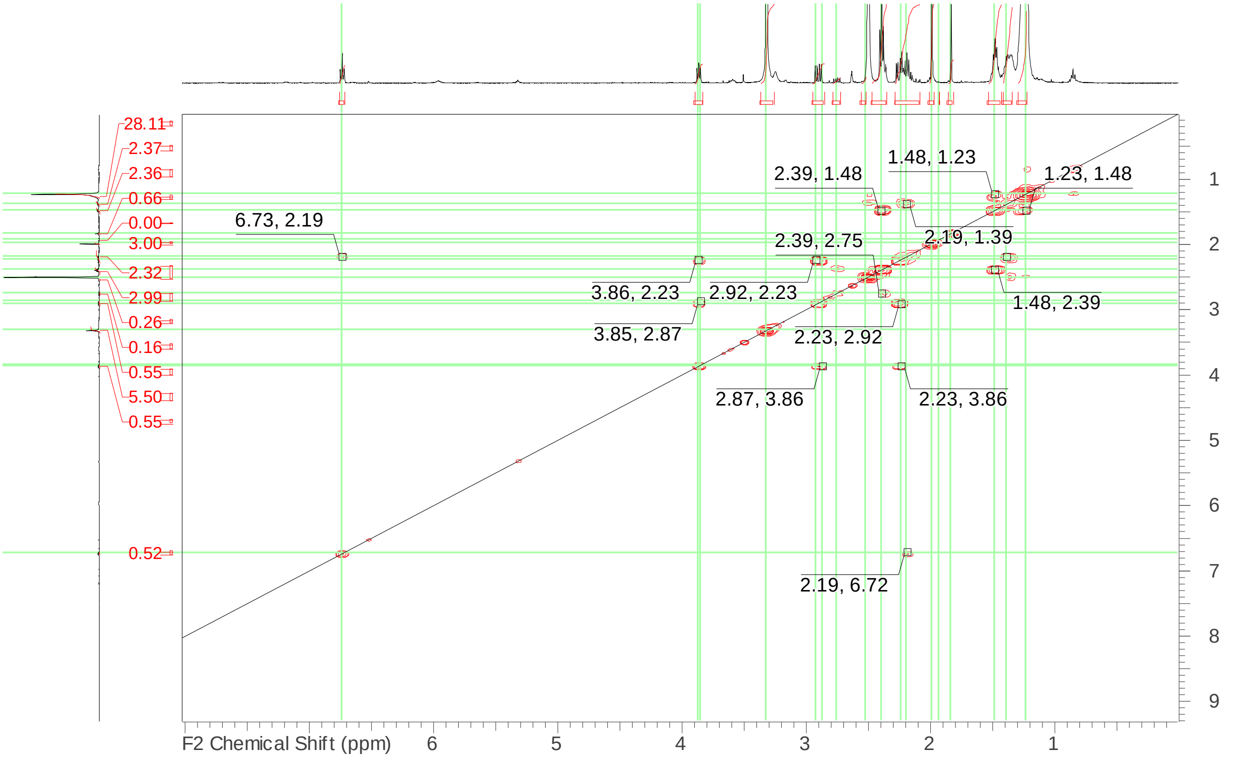


Figure S29: $^{1}$H, $^{1}$H COSY spectrum of skeletocutin O (**4**) in DMSO (500 MHz)

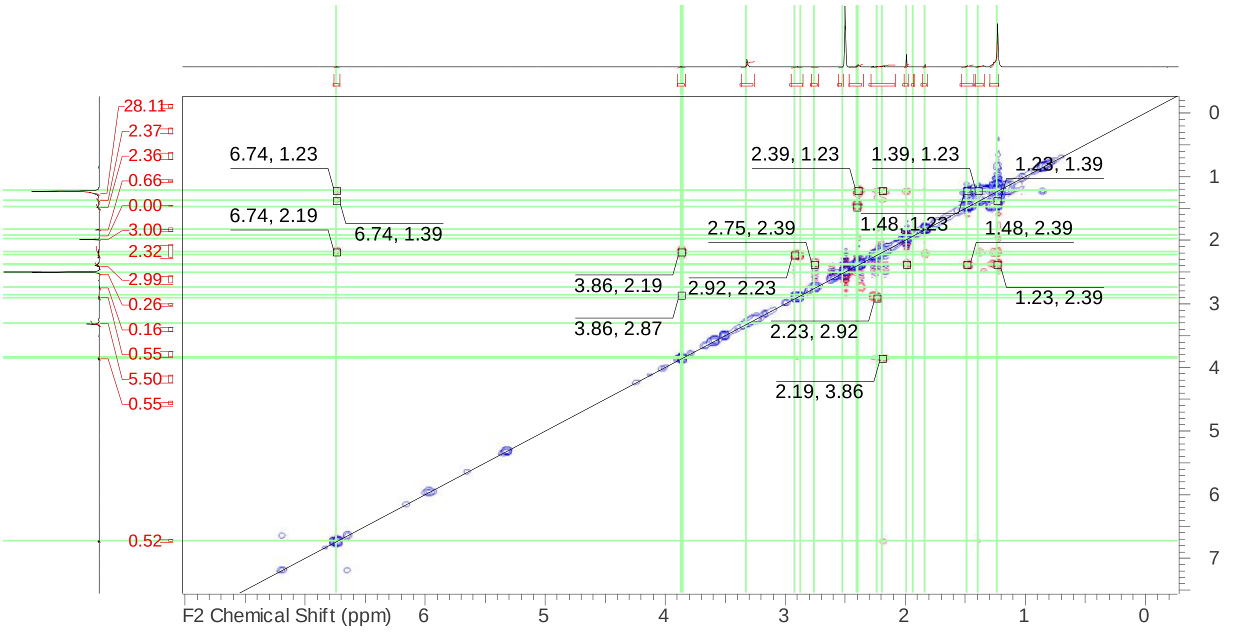


Figure S30: $^{1}$H, $^{1}$H ROESY spectrum of skeletocutin O (**4**) in DMSO (500 MHz)

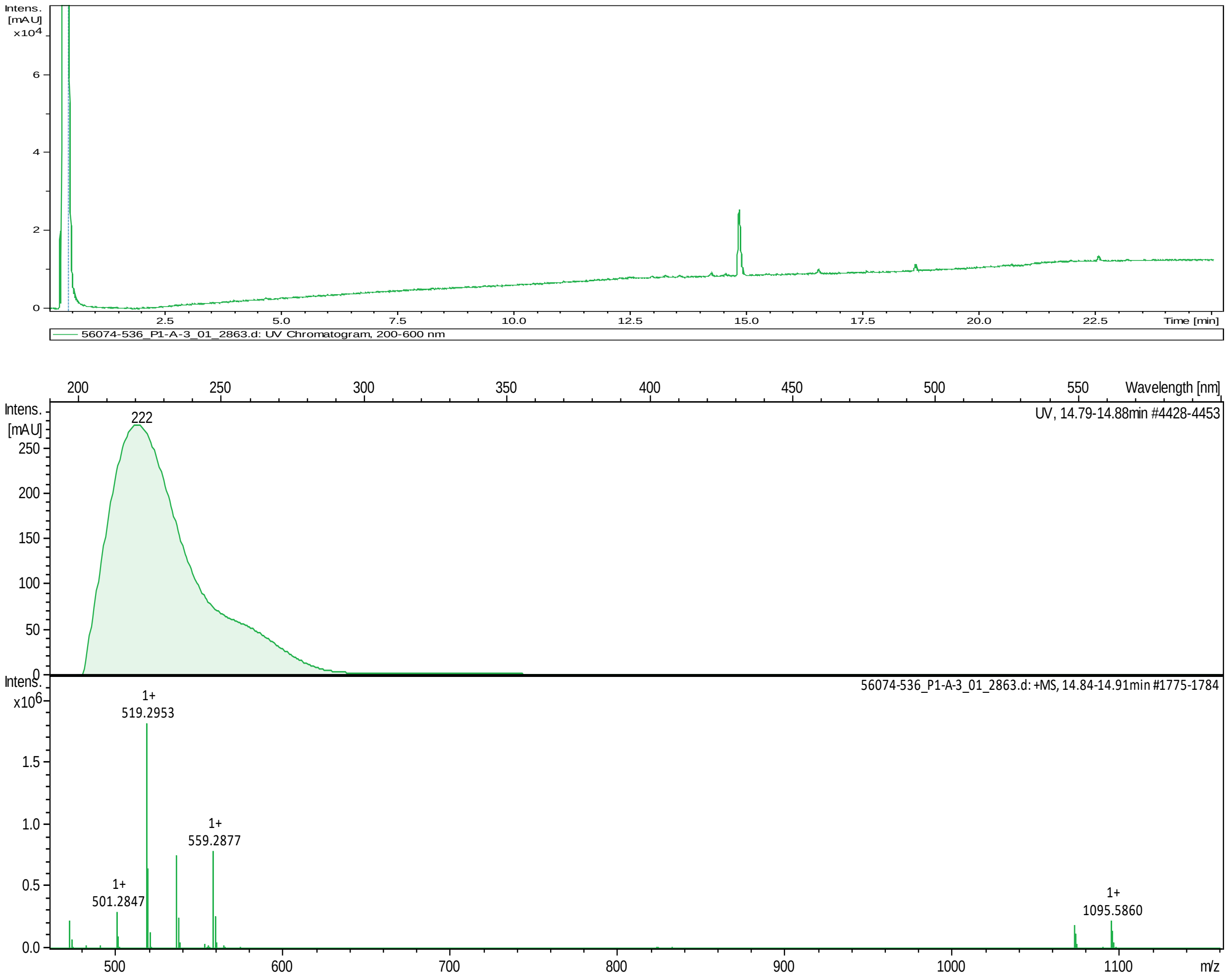


Figure S31: HRESIMS spectrum of skeletocutin P (**4**)

## 1 and 2D NMR data for skeletocutin Q (5)

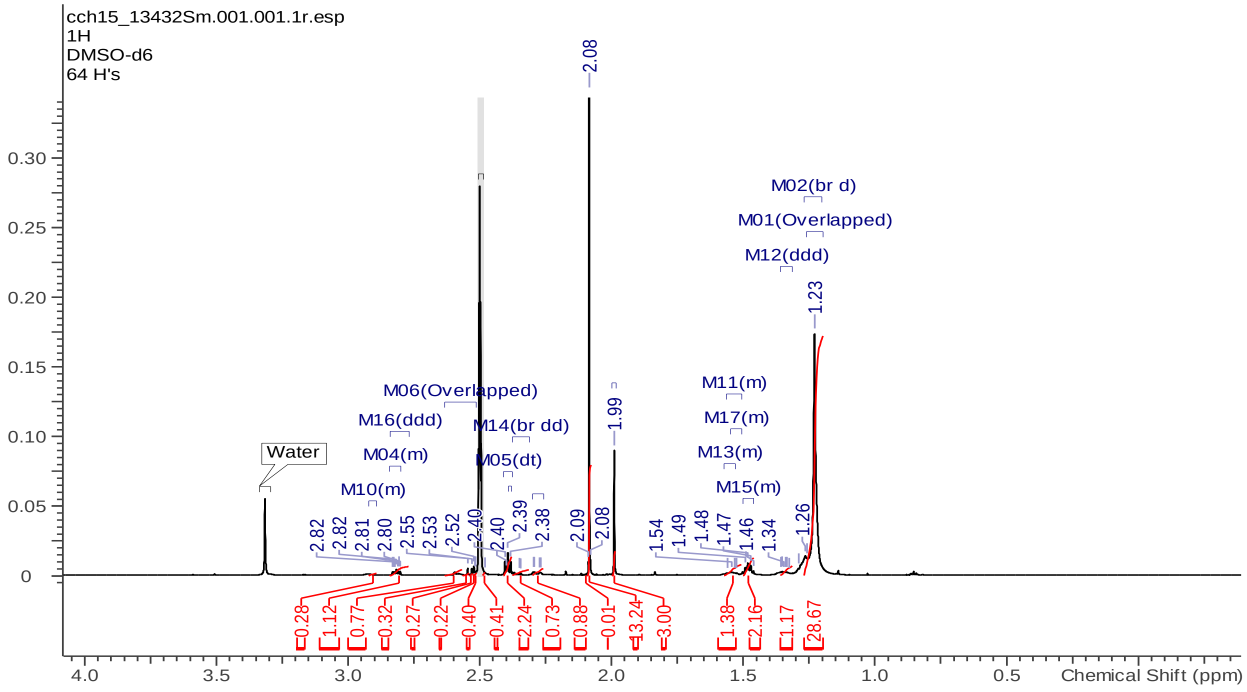


Figure S32: $^{1}$H NMR spectrum of skeletocutin Q (**5**) in DMSO (500 MHz)

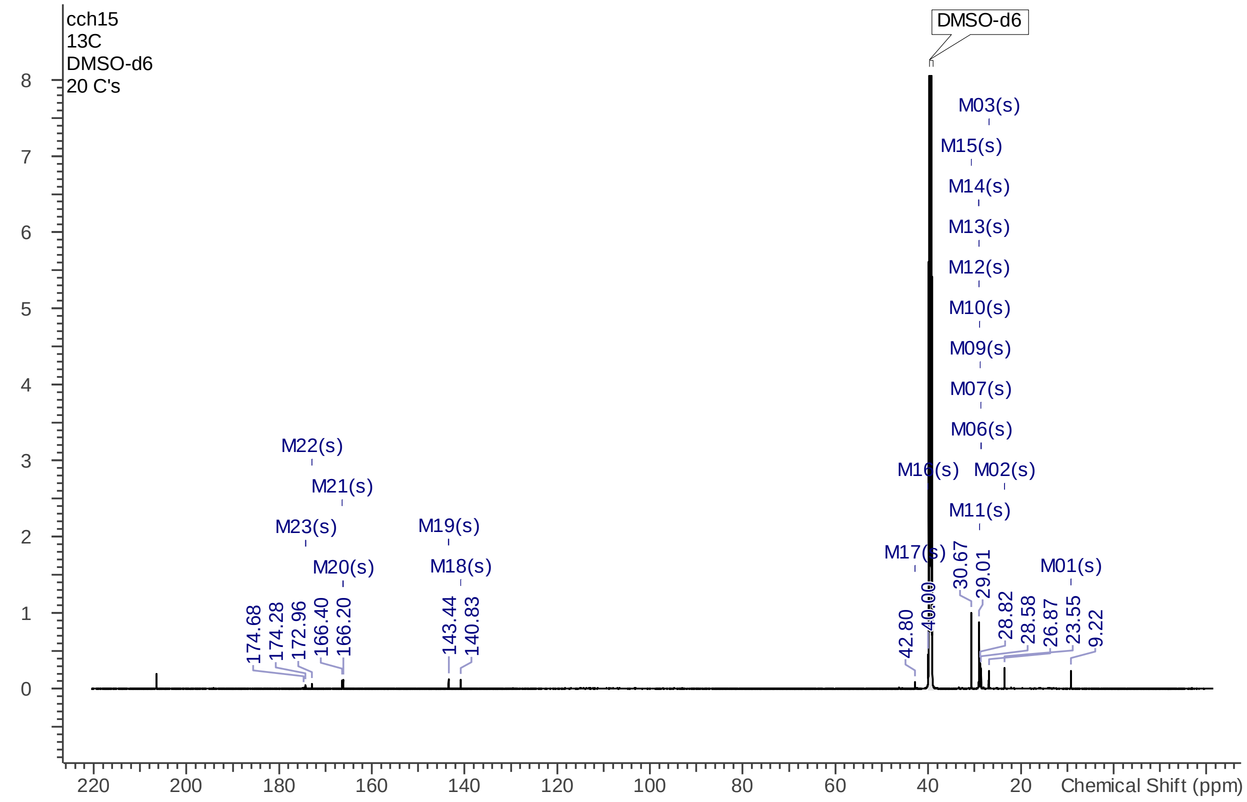


Figure S33: $^{13}$C NMR spectrum of skeletocutin Q (5) in DMSO (125 MHz)

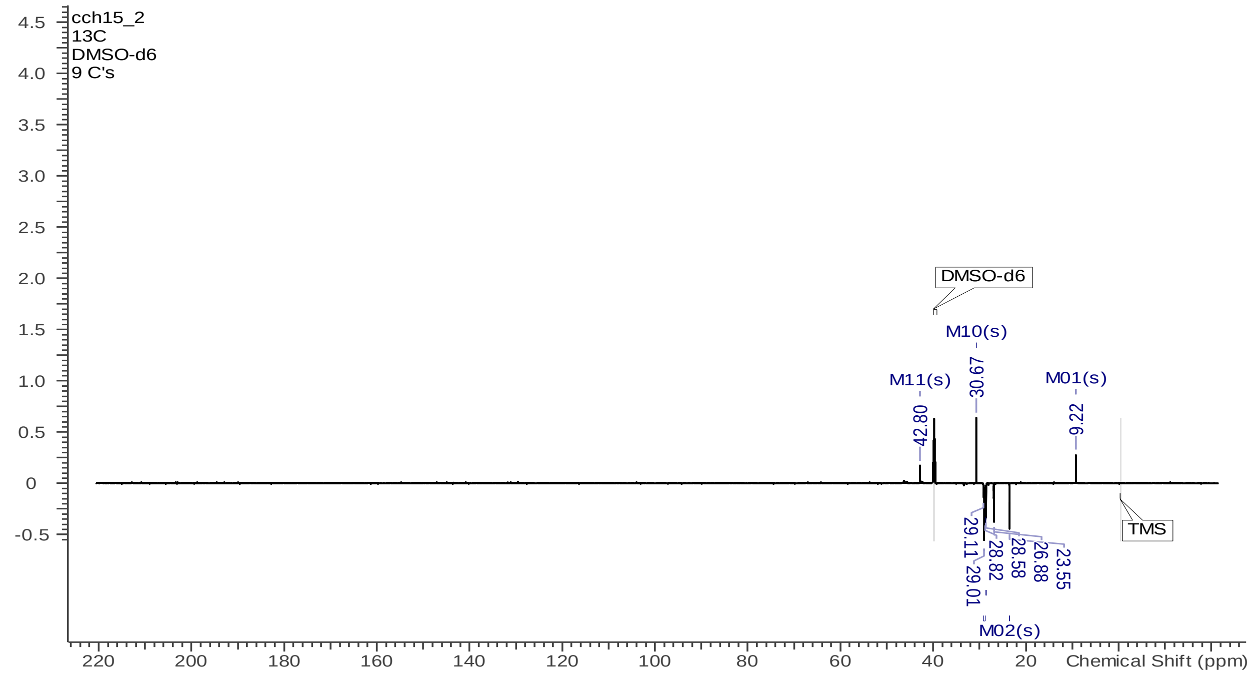


Figure S34: DEPT NMR spectrum of skeletocutin Q (**5**) in DMSO (125 MHz)

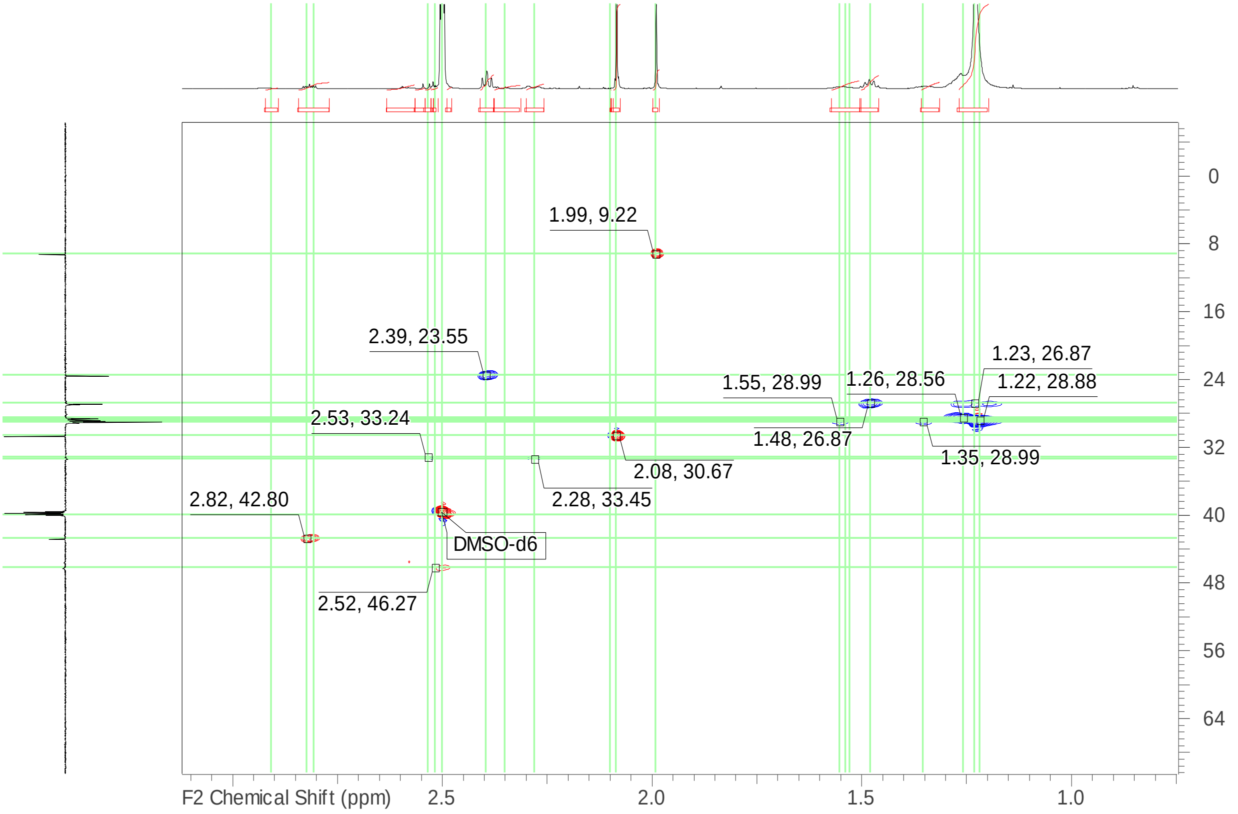


Figure S35: $^{1}$H, $^{13}$C HSQC spectrum of skeletocutin Q (**5**) in DMSO (500 MHz, 125 MHz)

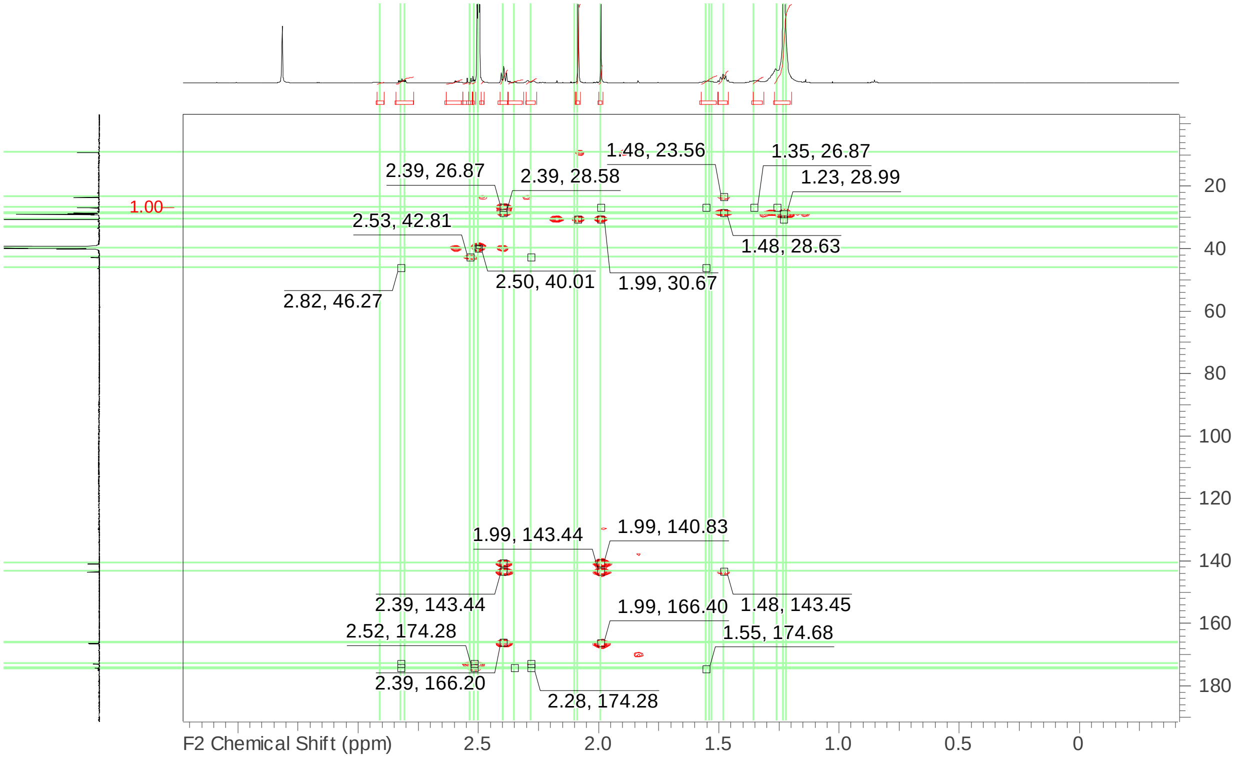


Figure S36: $^{1}H$, $^{13}C$ HMBC spectrum of skeletocutin Q (**5**) in DMSO (500 MHz, 125 MHz)

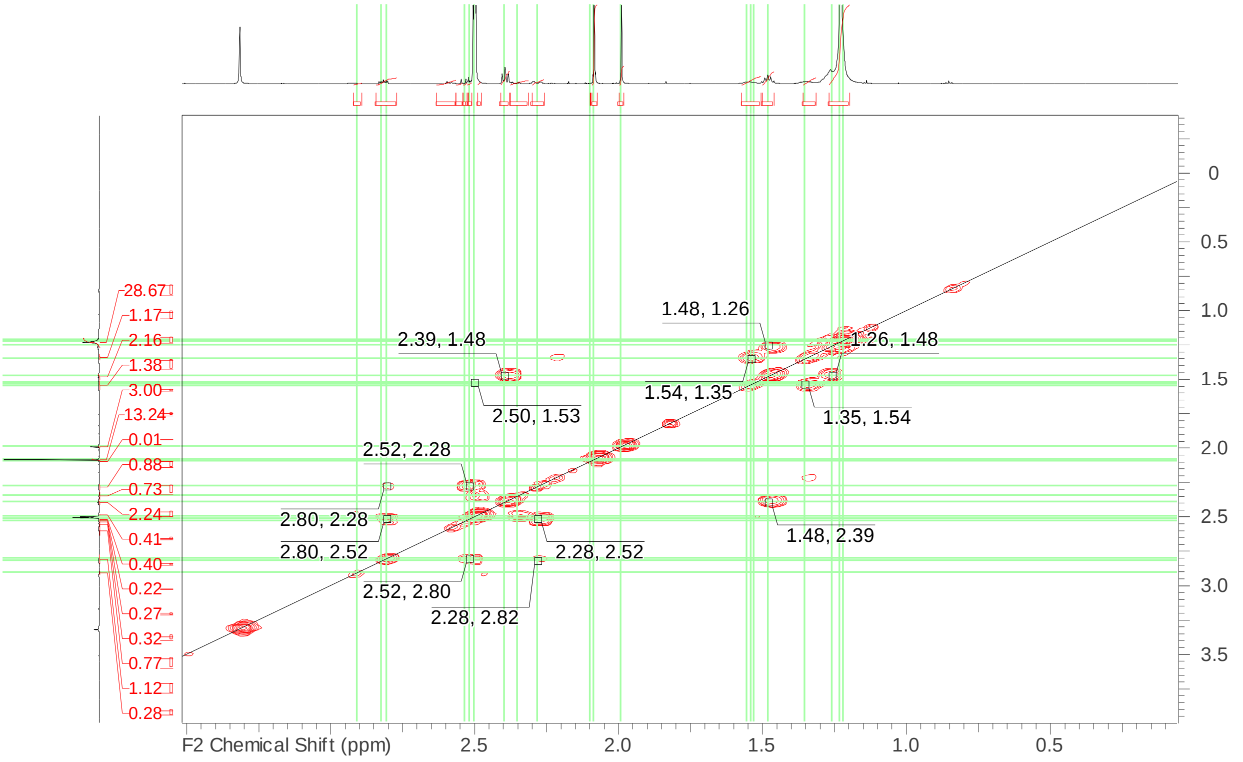


Figure S37: $^{1}H$, $^{1}H$ COSY spectrum of skeletocutin Q (**5**) in DMSO (500 MHz)

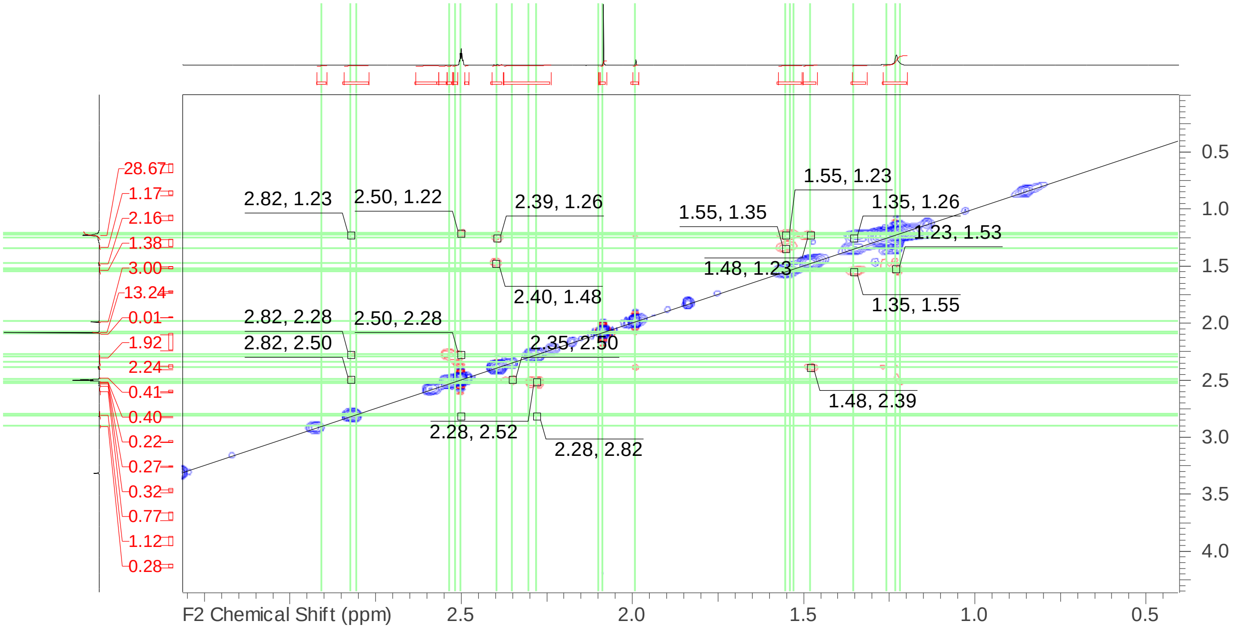


Figure S38: $^{1}H$, $^{1}H$ ROESY spectrum of skeletocutin Q (**5**) in DMSO (500 MHz)

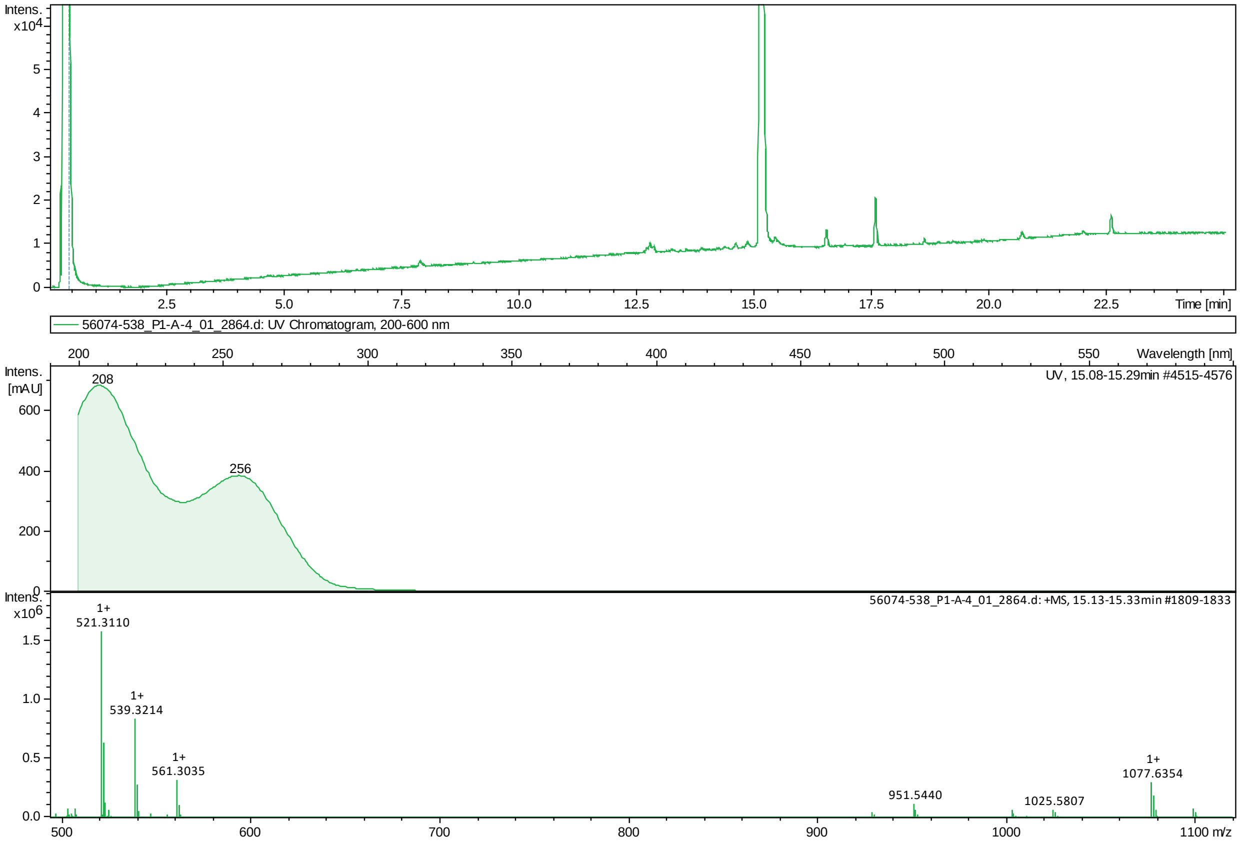


Figure S39: HRESIMS spectrum of skeletocutin Q (**5**)

## Media

YMG: 10 g/L malt extract, 4 g/L yeast extract, 4 g/L D-glucose and PH=6.3;

MHB: Mueller Hinton Broth (comprising beef infusion solids, 2.0 g/L; casein hydrolysate, 17.5 g/L; starch, 1.5 g/L).

## ITS sequence

>MUCL56074
ATATGCTTAAGTTCAGCGGGTAGTCCTACCCGATTTGAGGTGCAGATGTCAAAAGATTATTACAA
TCTGTCTTAAAAGACAACTAGAAGCGGAATTCCATACATGTGCTTAGACAGCTACAGCGTAGACA
ATTATCACACTGAAGCTAGACCTGAGCAAAGATTTCCAGCTAATATATTCAAGAGGAGCAGATTT
ATTACTAAACCTGCAAAGAGACCTCCAAATCCAAAGCACCAACATCATCAAAAAATGAAGAGGGC
TTTGAGAATACCATGACACTCAAACGGGCATGCCCTTCGGAATACCAAAGGGCGCAAGTTGCGT
TCAAAGATTCGATGATTCACTGAATTCTGCAATTCACATTACTTATCGCATTTCGCTGCGTTCTTC
ATCGATGCGAGAGCCAAGAGATCCGTTGCTAAAAGTTATATATAATGCGTTATTTAAGCGCAAGA
GACATTCATGATACAGCGTGTGTGAATGAAACATAGGAAGGCGTCAACAACTAGAGAGGAACCT
AAGTTCTTCTCCTGTATCAACCATCCTACAATATGTGCACAGGTGTTAAAGATGAGTTGGATTTGA
GCGAAGCGTGCACATGCCCCGAAAGGCCAGCTACAACTTCTTTCAAAGACTCGATAATGATCCT
TCCGCAGGTTCACCTACGGAAACCTTGTTACGACTTTTACTTCC

## Biofilm Inhibition

Table S1: biofilm inhibition results from plate reader

| compound \ concentration (µg/ml) | | 256 | 128 | 64 | 32 | 16 | 8 |
|---|---|---|---|---|---|---|---|
| **1** | replication 1 | 4.909 | 3.804 | 4.69 | 3.38 | 3.059 | 2.758 |
| | replication 2 | 4.54 | 3.615 | 3.419 | 3.45 | 3.706 | 3.282 |
| | replication 3 | 5.651 | 3.365 | 3.207 | 3.219 | 3.472 | 3.031 |
| | negative control (MEOH) | 4.373 | 3.631 | 4.202 | 3.617 | 3.224 | 3.538 |
| | | 4.205 | 4.216 | 3.215 | 3.357 | 3.421 | 3.643 |
| | | 4.569 | 4.594 | 3.798 | 3.462 | 4.358 | 4.125 |
| | positive control (Tetracycline) | 0.462 | 0.622 | 0.885 | 1.107 | 1.682 | 3.003 |
| **2** | replication 1 | 4.44 | 4.381 | 3.791 | 3.016 | 2.995 | 2.718 |
| | replication 2 | 4.364 | 3.14 | 2.979 | 3.176 | 2.916 | 3.048 |
| | replication 3 | 4.077 | 3.623 | 2.823 | 3.059 | 2.987 | 2.841 |
| | negative control (MEOH) | 3.958 | 3.476 | 3.269 | 3.055 | 3.192 | 3.003 |
| | | 3.818 | 3.113 | 3.222 | 3.538 | 3.349 | 2.896 |

| | | | | | | | |
|---|---|---|---|---|---|---|---|
| | | 4.795 | 3.576 | 3.266 | 3.384 | 3.755 | 3.142 |
| | positive control (Tetracycline) | 0.414 | 0.478 | 0.627 | 0.791 | 0.866 | 1.694 |
| **3** | replication 1 | 1.742 | 3.854 | 3.63 | 3.458 | 3.2 | 3.132 |
| | replication 2 | 2.26 | 3.496 | 3.041 | 2.875 | 2.837 | 2.88 |
| | replication 3 | 2.419 | 3.752 | 3.373 | 3.534 | 3.385 | 3.631 |
| | negative control (MEOH) | 6 | 4.205 | 3.706 | 3.401 | 3.513 | 3.95 |
| | | 4.457 | 4.265 | 3.583 | 3.446 | 2.956 | 3.677 |
| | | 4.196 | 3.562 | 3.427 | 3.183 | 2.892 | 3.137 |
| | positive control (Tetracycline) | 0.498 | 0.765 | 0.95 | 1.315 | 1.912 | 2.162 |
| **4** | replication 1 | 6 | 4.115 | 3.887 | 4.271 | 4.114 | 3.554 |
| | replication 2 | 4.841 | 4.224 | 3.524 | 4.702 | 4.47 | 4.243 |
| | replication 3 | 5.066 | 4.03 | 3.957 | 3.439 | 3.878 | 3.452 |
| | negative control (MEOH) | 3.116 | 4.203 | 4.277 | 4.279 | 3.787 | 4 |
| | | 3.732 | 3.278 | 3.656 | 3.817 | 3.901 | 3.565 |
| | | 4.3 | 3.534 | 4.155 | 4.417 | 4.138 | 4.731 |
| | positive control (Tetracycline) | 0.487 | 0.527 | 0.917 | 1.404 | 1.333 | 3.5 |
| **5** | replication 1 | 3.822 | 3.836 | 3.627 | 3.757 | 3.879 | 3.704 |
| | replication 2 | 3.988 | 3.597 | 3.623 | 4.452 | 3.633 | 3.444 |
| | replication 3 | 3.87 | 3.871 | 3.708 | 3.838 | 3.438 | 2.956 |
| | negative control (MEOH) | 4.884 | 3.244 | 3.509 | 4.237 | 3.885 | 3.846 |
| | | 4.562 | 6 | 3.695 | 3.348 | 3.258 | 3.626 |
| | | 5.044 | 5.319 | 3.951 | 4.112 | 4.127 | 6 |
| | positive control (Tetracycline) | 0.644 | 0.693 | 0.836 | 1.155 | 1.748 | 3.311 |
| **6** | replication 1 | 4.54 | 2.858 | 3.723 | 3.188 | 4.007 | 3.586 |
| | replication 2 | 6 | 2.819 | 3.478 | 3.328 | 3.612 | 3.766 |
| | replication 3 | 3.949 | 3.861 | 3.105 | 3.954 | 3.954 | 3.772 |
| | negative control (MEOH) | 3.045 | 3.272 | 4.255 | 3.937 | 4.245 | 4.124 |
| | | 3.147 | 3.757 | 4.497 | 3.871 | 4.101 | 3.952 |
| | | 3.079 | 4.935 | 4.338 | 4.485 | 4.872 | 3.874 |
| | positive control (Tetracycline) | 0.444 | 0.655 | 0.83 | 0.918 | 1.362 | 1.846 |
